# Tailoring diamondised nanocarbon-loaded poly(lactic acid) composites for highly electroactive surfaces: extrusion and characterisation of filaments for improved 3D-printed surfaces

**DOI:** 10.1007/s00604-023-05940-7

**Published:** 2023-08-28

**Authors:** Mateusz Cieślik, Agnieszka Susik, Mariusz Banasiak, Robert Bogdanowicz, Krzysztof Formela, Jacek Ryl

**Affiliations:** 1 Department of Analytical Chemistry, Faculty of Chemistry, University of Gdańsk, Wita Stwosza 63, 80-308 Gdańsk, Poland; 2 Division of Electrochemistry and Surface Physical Chemistry, Faculty of Applied Physics and Mathematics, Gdańsk University of Technology, Gabriela Narutowicza 11/12, 80-233 Gdańsk, Poland; 3 Department of Polymer Technology, Faculty of Chemistry, Gdańsk University of Technology, Gabriela Narutowicza 11/12, 80-233 Gdańsk, Poland; 4 Department of Metrology and Optoelectronics, Faculty of Electronics, Telecommunication and Informatics, Gdańsk University of Technology, Gabriela Narutowicza 11/12, 80-233 Gdańsk, Poland

**Keywords:** Material extrusion, Diamondised nanocarbons, 3D-printable filament, Dopamine detection, Electrochemical analysis, Differential pulse voltammetry

## Abstract

A new 3D-printable composite has been developed dedicated to electroanalytical applications. Two types of diamondised nanocarbons - detonation nanodiamonds (DNDs) and boron-doped carbon nanowalls (BCNWs) - were added as fillers in poly(lactic acid) (PLA)-based composites to extrude 3D filaments. Carbon black served as a primary filler to reach high composite conductivity at low diamondised nanocarbon concentrations (0.01 to 0.2 S/cm, depending on the type and amount of filler). The aim was to thoroughly describe and understand the interactions between the composite components and how they affect the rheological, mechanical and thermal properties, and electrochemical characteristics of filaments and material extrusion printouts. The electrocatalytic properties of composite-based electrodes, fabricated with a simple 3D pen, were evaluated using multiple electrochemical techniques (cyclic and differential pulse voltammetry and electrochemical impedance spectroscopy). The results showed that the addition of 5 wt% of any of the diamond-rich nanocarbons fillers significantly enhanced the redox process kinetics, leading to lower redox activation overpotentials compared with carbon black–loaded PLA. The detection of dopamine was successfully achieved through fabricated composite electrodes, exhibiting lower limits of detection (0.12 μM for DND and 0.18 μM for BCNW) compared with the reference CB-PLA electrodes (0.48 μM). The thermogravimetric results demonstrated that both DND and BCNW powders can accelerate thermal degradation. The presence of diamondised nanocarbons, regardless of their type, resulted in a decrease in the decomposition temperature of the composite. The study provides insight into the interactions between composite components and their impact on the electrochemical properties of 3D-printed surfaces, suggesting electroanalytic potential.

**Graphical abstract:**

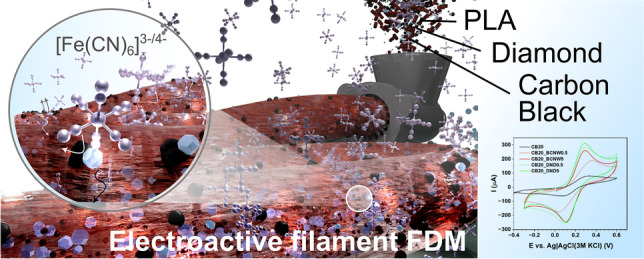

**Supplementary Information:**

The online version contains supplementary material available at 10.1007/s00604-023-05940-7.

## Introduction

The immense popularity of three-dimensional (3D) printing in various fields of science and industry [1–3] has led to the development of new specific-purpose, dedicated composite materials. In the case of material extrusion (ME), also known as fused deposition modelling (FDM®) technology, thin filaments are used to deliver materials for printing objects. One of the most popular filament materials used for ME is poly(lactic acid) (PLA), whose attractiveness originates from its enhanced mechanical properties, biodegradability and ease of printing among other things [4, 5]. Specific functional properties are achieved by adding micro- or nanofillers to the polymer matrix. For example, Antoniac et al. [6] created a PLA-based composite with magnesium particles and vitamin E which was used to print a front cross ligament screw and was characterised by biocompatibility and structural integrity. Here, the vitamin E addition also ensured the adhesion of Mg particles to the PLA matrix. Conductive carbon-based composites with a PLA matrix have seen dynamic development in recent years and found many applications in electrochemical energy storage, water splitting and electroanalysis [7–9].

The conductivity of 3D printable composites is most often obtained through the addition of different forms of conductive carbon nanofillers, such as graphene, carbon black (CB) or carbon nanotubes (CNTs). The amount of these nanofillers must be sufficient to form continuous conductive paths within the composite volume according to percolation theory [10]. Uniform distribution of conductive paths is key to obtaining electrically homogeneous electrode surfaces and avoiding the effect of partially blocked electrode areas [11]. One should note that the thermal stability of the filament under reprocessing is another concern, which might decrease the electrochemical performance by nanofiller agglomeration and so a decrease in the number of conductive paths [12]. Notably, while conductivity enhancement is similar and depends on the formation of conductive paths within the material, the electrochemical response of these composites differs significantly [13, 14].

In electroanalysis, the bulk of the currently available reports utilise commercially available conductive filaments—Proto-pasta® [14–17], and Black Magic 3D® [14, 18, 19]—using carbon black and graphene as conductive fillers, respectively. It should be noted that these filaments were most likely not designed for electrochemical applications. Moreover, Black Magic 3D® is no longer available on the market, which makes it impossible to continue the research conducted with this material. The possibility of tailoring the functional properties and lack of commercial availability of filaments are the fundamental arguments in favour of creating one’s own base of composite materials dedicated to 3D printing for electrochemical applications. In this regard, Foster et al. [20] fabricated filament containing 25 wt% of nanographite in a PLA matrix, which was further successfully used for Pb and Cd ion detection in water, with a limit of detection (LOD) equal to 2.0 and 2.2 μg/L, respectively. Stefano et al. [21] conclude that an even higher graphite content of up to 40 wt% may be used effectively. 3D-printed electrodes, after surface modification, were capable of detecting the SARS-CoV-2 virus in trace concentrations (0.10 μg/mL). The same material was also used by the authors for the analysis of paraquat and carbendazim in food and water samples [22]. Carbon-based fillers have also been applied in other polymers utilised in ME technology such as thermoplastic polyurethane (TPU). Answer et al. [23] reported that a TPU matrix with multiwall carbon nanotubes reveals a positive impact on the hydrophobic properties, increasing the contact angle to 30% and lowering the strain by ~99% during mechanical tests. On the other hand, the presence of boron nitride nanosheets nearly doubles the tensile strength and results in 6.5-fold higher thermal conductivity compared to the substrate [24]. Importantly, anisotropic properties in the electrical impedance of materials made by extrusion-based additive manufacturing were reported by Daniel et al. [25], which were ascribed to the layer-by-layer nature of the process.

Due to their unique properties, such as high hardness, mechanical, chemical and thermal stability, biocompatibility and antifouling, diamondised nanocarbons have found application in implantology [26], where selective laser melting technology is often used for layer-by-layer sintering of diamond powders [27]. Thermoplastic polymers with high-pressure high-temperature microdiamond fillers were used in ME to create flow injection analysis systems [28] and printed heat sinks [29]. The goal of using diamonds in an acrylonitrile butadiene styrene (ABS) matrix was aimed at increasing thermal conductivity and mechanical strength. It should be noted that a commercially available PLA filament with nanodiamond fillers (electrically insulating) was developed (uDiamond® Vox P by Carbodeon) and tested in medical devices [26]. In another approach, the same filament was carboxylated, and the obtained nano-sized diamonds were used in rubber-based composites [30]. Furthermore, composite diamond coatings have already been applied to substrates with various additive manufacturing methods as directed energy deposition—laser-based technology [31], cold spraying of micropowders [32] and ME technology utilising a geopolymer matrix for lowered viscosity and improved thermal conductivity [33].

Kalsoom et al. [34] developed a composite with conductive boron-doped diamond (BDD) and LiCl in an ABS matrix, which was successfully used to 3D print an accurate humidity sensor. Here, high BDD loading (60 wt%) was used to achieve electric conductivity of the material, while the sensing performance was obtained by the LiCl, which is broadly used in humidity sensors. Regretfully, the material was only investigated for its electric and not electrochemical properties, primarily considering to be unique properties of BDD in terms of electrochemical applications. The above-stated mechanical and chemical stability, but also wide electrolytic window, low charge transfer resistances at the electrode/electrolyte interphase and low background currents, put BDD and other diamond-based materials among the most efficient materials for electroanalysis. In a previous study, we investigated a BDD-conductive PLA junction, revealing that BDD foils decorating the surface of graphene-PLA printouts created a rigid and mechanically stable electrode interface with enhanced local tunnelling in the diamond/graphene junction configuration [35]. The electrode was effectively used for the detection of explosives, where the 2,4,6-trinitrotoluene (TNT) LOD was as low as 87 ppb.

Forming BDD foils is a complex process that is hard to scale up; however, the extraordinary electroanalytical results from this work motivated us towards the development of the first 3D-printable filament in ME technology with diamondised nanocarbon powder fillers, dedicated to electroanalytical purposes.

We have developed the first conductive, PLA-based composite loaded with diamondised nanocarbons (DNCs) and carbon black, which was fabricated in the form of a 3D-printable filament and was dedicated to electroanalytic applications. Two DNC types were investigated in this work as fillers for PLA-based composites, namely detonation nanodiamonds (DNDs) and boron-doped carbon nanowalls (BCNWs). The study unveils the impact of DNCs and CB on the rheology and thermo-mechanical properties of the PLA composite and their potential to enhance charge transfer within the electrode/electrolyte interface for improved electrochemical performance. Experiments showed that introducing DNCs at low concentrations (up to 5 wt%) enhances electrocatalytic performance by ensuring linear diffusion within the densely overlapping diffusion layers [11, 36] surrounding the uniformly distributed DNCs on the electrode surface. The addition of CB facilitates percolation paths, ensuring high electrical conductivity. Furthermore, this research delivers a thorough description and understanding of the interactions between composite components and mechanisms and how they affect the rheological, mechanical and thermal properties.

## Material and method

### Materials

#### Polymer matrix

Poly(lactic acid) PLA Ingeo Biopolymer 3D450 was produced by Natureworks. According to the technical data sheet provided by the producer, the monofilament made with Ingeo 3D450 offers an optimum balance between adhesion and ease of removal from the build substrate. Moreover, the technical data sheet indicates that the typical physical properties of PLA 3D450 are specific gravity of 1.32 g/cm^3^, an MFR_210°C/2.16kg_ of 18–26 g/10 min (our results show 15.1±0.4 g/10 min) and a melting temperature of 165–180°C.

#### Conductive carbon fillers

Conductive carbon black with the tradename Ensaco 250G was purchased from Imerys Graphite & Carbon Switzerland Ltd. (Bironico, Switzerland). According to the data sheet provided by the producer, Ensaco 250G is characterised by a BET nitrogen adsorption surface area estimated at 66 m^2^/g (our results show 49 m^2^/g), an oil absorption number of 194 ml/100 g and a total sulphur content of 120 ppm.

#### Diamondised nanocarbon fillers

DNDs, typically 5–10 nm in diameter, are formed as a result of a controlled explosion with TNT or another suitable explosive [37], making their structure heavily contaminated with nitrogen atoms even after cleaning [38]. As a result, increased carrier concentration and higher charge transfer rates are achieved [39]. DND particles were supplied by Adamas Nanotechnologies Inc., Raleigh, NC, USA. According to the producer, the DND’s Z-average size is 200 nm, the zeta potential is −15 mV and the ash content is 2 wt%.

BCNWs are highly characteristic carbon structures in the form of walls aligned vertically to the substrate, rich in both sp^2^ and sp^3^ phases and heavily doped with boron [40, 41]. During electroanalysis, BCNWs exhibit outstanding properties: high standard rate constant (*k*_0_), high electrocatalytic activity [42] and biosensor sensitivity [43], photocatalytic capabilities [44] and superior redox performance [45, 46]. BCNWs were deposited on micron-sized glassy carbon (GC) powder (SIGRADUR® G, Germany) with a Microwave Plasma Assisted Chemical Vapour Deposition (MPACVD) system (SEKI Technotron AX5400S, Japan). Chemically stable GC was used as a substrate for BCNW to fix the average diameter of the final particles formed during the CVD process and to ensure the high kinetics of the nanowall nucleation. The formation of freestanding BCNW nanoparticles without a substrate is inefficient and results in the growth of random inhomogeneous particle shapes. First, 150 mg of glassy carbon was distributed evenly on a molybdenum cylinder using a small amount of isopropanol, which evaporated under a stable N_2_ flux after distribution. The BCNWs were deposited in a MPACVD chamber for 3 h with the stage heated up to 850°C, microwave radiation power set to 1300 W and pressure to 50 Torr. The gas used was a mixture of H_2_, CH_4_, B_2_H_6_ and N_2_ with a total gas flow of 303 sccm. B_2_H_6_, as a boron dopant, was set to a 2000 ppm [B]/[C] ratio, and the CH_4_/H_2_ ratio was kept at 1%.

#### Reagents

Solutions for electrochemical measurements were prepared using deionised water of resistivity not less than 18 MΩ. Potassium ferricyanide and ferrocyanide (99% w/w), sodium hydroxide (98% w/w) and potassium chloride (99% w/w) were purchased from Sigma-Aldrich.

### Fabrication of conductive composites

**Fig. 1:**
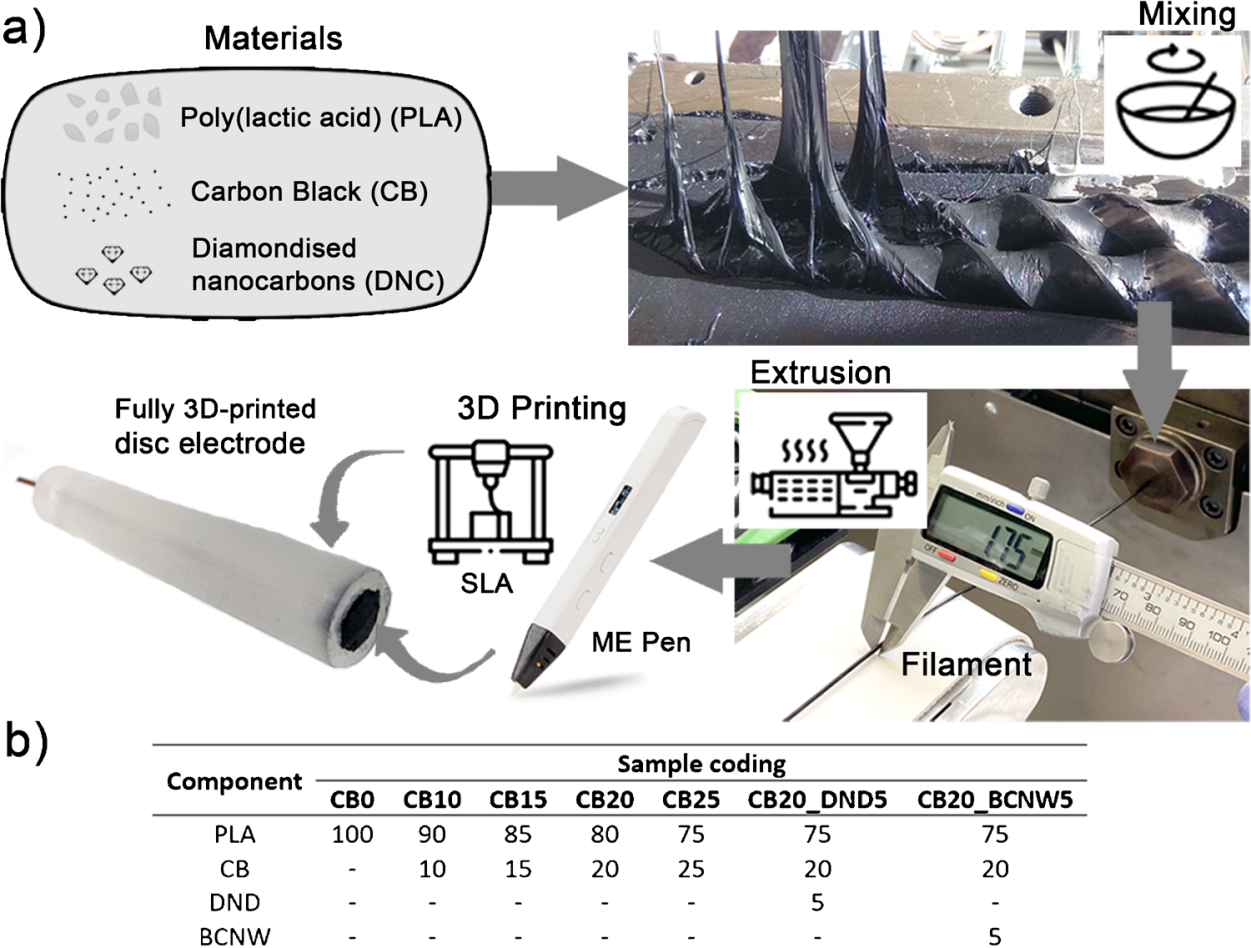
**a** Schematic representation of the consecutive steps of filament production and fabrication of fully 3D-printed disc electrode; **b** composition of the conductive 3D printable filaments fabricated and sample coding used in the manuscript

CB-PLA and CB-DNC-PLA (DNC: DND or BCNW) composites were prepared by melt-compounding using a model 2T30-16 laboratory conical twin screw extruder produced by Łukasiewicz Research Network–Institute of Engineering of Polymer Materials and Dyes (Toruń, Poland). The barrel of the 2T30-16 microcompounder is equipped with a bypass, so samples can be produced by continuous or batch mixing. PLA and carbonic fillers (CB or CB+DNC) were mixed by circulation in bypass mode for 8 min, while the screws’ rotation speed was 100 rpm. Temperature of the extruder barrel from the hopper to the die was 140, 170 and 200°C at each of three heating/cooling zones. Subsequently, the vessel was switched to an extruder die with a diameter 1.75 mm, the extruded material was cooled by air and 3D filaments made from the prepared composites were formulated. The sample preparation protocol and sample coding of the composites are summarised in Fig. 1.

### Methodology

#### Physico-chemical studies of the fillers

A Bruker D2 Phaser diffractometer with Cu Kα radiation (λ = 1.54056 Å) and an XE-T detector were used to carry out X-ray diffraction (XRD) measurements. A 2θ range of 5–70° was used to collect the data. High-resolution X-ray photoelectron spectroscopy (XPS) measurements were carried out using an Escalab 250Xi spectroscope from Thermo Fisher Scientific. The X-ray source was AlKα and the spot was 500 μm. The pass energy was set to 20 eV. Charge compensation was assured through low-energy electron and Ar^+^ ion bombardment of the sample, with final peak calibration at adventitious carbon (*C 1s* at 284.6 eV). Specific surface areas of the fillers were measured by nitrogen adsorption at 77 K (NOVAtouch™ 2, Quantachrome Instruments) and calculated using the BET linear equation in the approximate relative pressure range of 0.1 to 0.3. The correlation coefficient of the linear regression was not less than 0.99. Prior to the measurements, the samples were degassed under vacuum at 40°C for 12h.

#### Surface morphology analysis

A scanning electron microscope (SEM) was used to study the topography of the nanocarbon fillers and fabricated composites. The measurements were carried out using an FEI Quanta 250 FEG instrument (Thermo Fisher Scientific) equipped with a Schottky field emission gun, operating at an accelerating voltage of 5 kV.

#### Physico-chemical properties of the composites

A silicon plate was used to perform the measurements for the DNDs and BCNW. Fourier transform infrared spectroscopy (FTIR) analysis of the PLA-based composites was performed using an IRTracer-100 from Shimadzu. The device was equipped with a single-reflection ATR accessory with a prism made from germanium crystal. Measurements were performed in attenuated total reflectance mode, at 4 cm^−1^ resolution and 45 scans in the range 4000–650 cm^−1^. An XploRA Plus spectrometer from Horiba Scientific was used for Raman spectroscopy analyses. A 532-nm laser was used and the spectral acquisition time was 10 scans at 15 s/scan. The spectra were collected within the region from 200 to 3400 cm^−1^. The filaments were broken in half directly prior to their measurement and studied in the cross-section area. For each tested sample, 5 spots distributed over the examined surface were tested to ensure the representativeness of the results.

#### Electric properties of the composites

The four-probe DC conductivity measurements were performed in preliminary studies, using four crocodile clips as probes connected to the source-measure unit (Keithley 2450, Tektronix) responsible for generating the electrical current and measuring the voltage drop. The distance between the inner probes was fixed at 100 mm during the measurements in three different spots and the resistance was calculated according to Ohm’s law, using 100 μA of electrical current flow. The resistivity was calculated by considering the length and diameter of the sample. Consequently, the AC conductivity of the selected samples was studied with broadband dielectric spectroscopy (BDS). Measurements were carried out using a Novocontrol Concept 40 Alpha-A with a ZG4 dielectric interface, using an AC voltage of 1 V_rms_ in a frequency range from 10 mHz to 1 MHz. The temperature range was selected to coincide with the materials’ scope of work, with a cooling and heating temperature step of 5°C, ranging from 0 to 40°C. Gold electrodes were evaporated in a vacuum at the polished plane parallel surfaces of the samples for the electrical measurements. The samples were approximately 2 mm thick and 10 mm in diameter. Measurements were taken under a nitrogen atmosphere using a Quatro Cryosystem temperature-controlling system.

#### Thermomechanical and rheological measurements

A thermogravimetric analysis (TGA) was performed using a Netzsch TG 209 apparatus using approx. 10 mg samples in the temperature range 25–800°C and under a nitrogen atmosphere at a heating rate of 10°C/min. A differential scanning calorimetry (DSC) measurement was carried out on a DSC 204 F1 Phoenix apparatus from Netzsch Group. The glass transition temperature (*T*_g_), melting point (*T*_m_) and crystallisation temperature (*T*_cc_) of the studied samples were investigated in the temperature range of 20–220°C under a nitrogen atmosphere at a heating rate of 10°C/min. The melt mass-flow rates (MFRs) of the PLA-based composites were investigated using a Zwick mFlow plastometer (Ulm, Germany) according to ISO 1133 at 210°C, with a load of 2.16 kg. For the samples whose MFR parameter was impossible to measure at 210°C and a load 2.16 kg, a different load or smaller capillary die diameter was applied. For those samples, a note is provided in the text. For selected samples, a dynamic mechanical analysis (DMA) was performed using a TA Instruments DMA Q800 (New Castle, DE, USA), in single cantilever bending mode from 0 to 180°C at a testing frequency of 1 Hz. A heating rate of 4°C/min was applied to the samples with dimensions of 40 × 10 × 2 mm.

#### Electrode fabrication

The produced composites were extruded in the form of filaments and used to print the conductive part of a disc electrode using a 3D Pen PRO printer (Mynt3D, USA). A similar approach was found to be effective elsewhere [47]. The printing temperature was 230°C. The electrode surface area was 0.4 cm^2^. The non-conductive body of the disc electrodes was prepared using 3D technology (vat photopolymerisation, using UV-curing resin). A copper plate with a wire was used to assure electrical contact. The utilised setup is illustrated in Fig. 1 and in the SI file, Fig. S1. This method is excellent for testing new materials for electrochemical purposes, because it does not need large amounts of filament for testing.

#### Electrochemical studies

The electrocatalytic properties of the fabricated composites were evaluated in a three-electrode setup with Ag/AgCl/3M KCl reference electrode and platinum mesh counter electrode. Electrode evaluation was carried out using cyclic voltammetry (CV), differential pulse voltammetry (DPV), and electrochemical impedance spectroscopy (EIS). First, the electrochemical activation procedure has to be carried out in 1M NaOH, using the methodology stated elsewhere [12, 48]. See the SI file, section S2 for more details. Next, an electrode kinetics assessment was carried out using 1 mM [Fe(CN)_6_]^3-/4-^ redox probe in 0.1 M KCl. The samples were pre-conditioned in the electrolyte for 10 min prior to the experiment. CVs were registered with a 100 mV/s scan rate in different polarisation ranges, sufficient to record the [Fe(CN)_6_]^3-/4-^ oxidation/reduction peaks. The EIS studies were carried out under formal potential, *E*_F_, and in the frequency range 1 Hz to 45 kHz. The electrodes were studied using DPV towards dopamine detection (0.9 to 1000 mM range) in 0.05 M phosphate buffer solution (PBS), pH = 4.5. The DPV scan rate was 5 mV/s. These measurements were performed using a VSP-300 potentiostat from BioLogic.

## Results and discussion

### Carbon black’s impact on thermomechanical properties of composite and conductive path formation

**Fig. 2:**
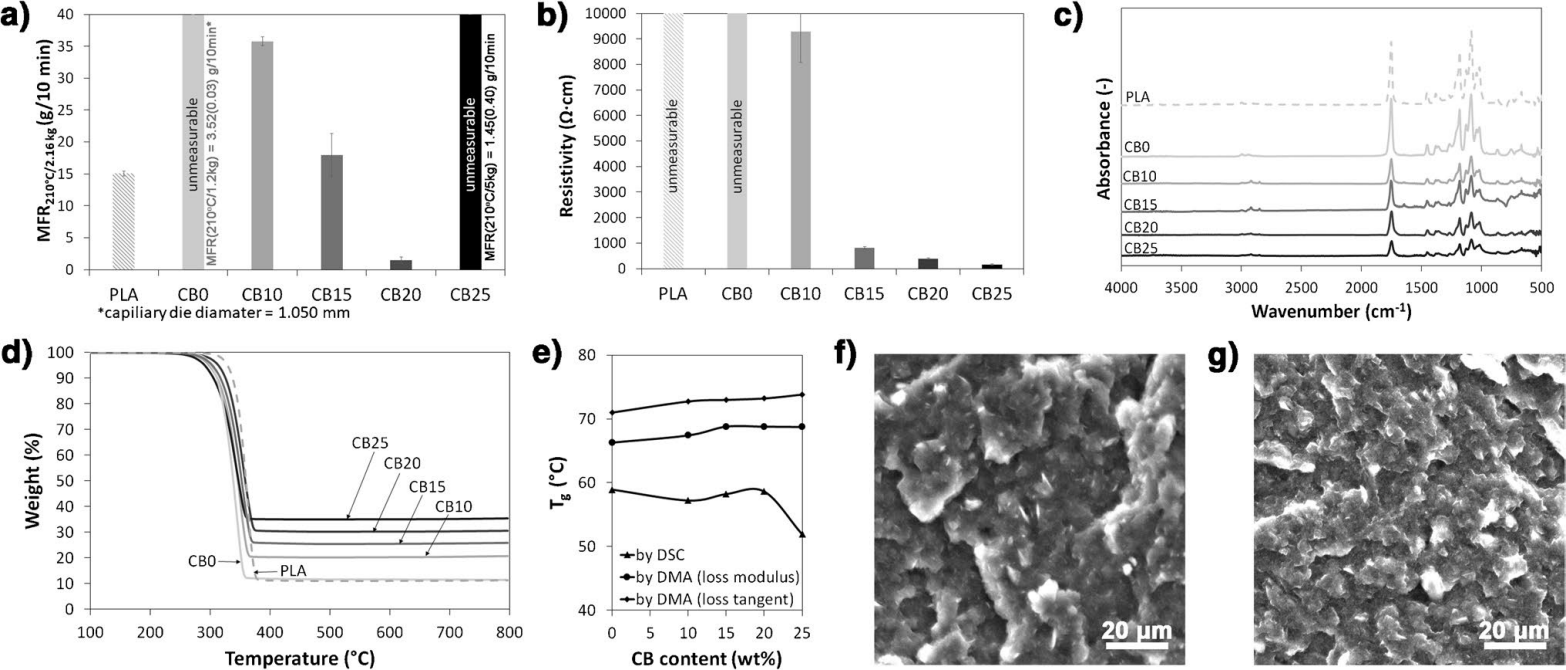
Processing, thermal and structural properties of CB-PLA composites with different CB contents: **a** MFR, **b** electrical resistivity, **c** FTIR, **d** TGA, **e** glass transition temperature *T*_g_, **f**–**g** SEM topography for **f** CB10 and **g** CB25 composite surface

According to the previously stated hypothesis, the amount of diamondised nanocarbon filler does not need to be high to assure enhanced electrolytic properties. Therefore, finding the optimal carbon black load in the composite and understanding its direct effect not only on the electric properties but also on the structural and thermomechanical characteristics constitutes the first key aspect of our research. Figure 2a presents the results of the melt flow studies used to determine the processability and rheological behaviour of CB-PLA composites with different CB amounts (10–25 wt%). The MFR is correlated to the viscosity of thermoplastics and indirectly with the molecular weight of polymers, which provides information about the overall performance properties of the thermoplastic materials. The obtained results indicate that the process of compounding by extrusion causes degradation of the PLA, which meant that it was impossible to examine the MFR for sample CB0 (PLA after extrusion without CB filler) in the studied conditions (210°C, 2.16 kg). The flowability of sample CB0 was much higher compared to PLA granulate without thermomechanical properties. Therefore, to perform the MFR measurement for CB0, a capillary die with a diameter of 1.050 mm and a load reduced to 1.2 kg were applied. Amorin et al. [49] showed that elevated temperature and shear forces act on PLA during extrusion and also during MFR measurements, resulting in thermodegradation of the PLA, which is governed by scission reactions. Increasing the amount of filler in the material increases the viscosity of the plastic and thus reduces the degree of mobility of the polymer chains [50]. Therefore, it should be pointed out that controlled degradation of PLA represents a promising approach to the preparation of highly filled PLA-based composites, especially for materials dedicated to ME 3D printing.

As could be predicted for CB-PLA composites, when increasing the amount of CB filler, the measured MFR value decreases, and for the CB25 sample, the measurement of the melt flow rate in the studied conditions (210°C, 2.16 kg) was impossible. The MFR for the CB25 sample was determined at 210°C, but with of a higher load of 5 kg. According to Wang et al. [51], an MFR of around 10 g/10 min (load: 2.16 kg; ISO 1133) is put forward for successful printing (190–220°C), which enables fast and practical screening of PLA-based materials. Considering these criteria for the studied CB-PLA composites, acceptable flowability was attained for samples up to 20 wt%. It should be mentioned that the content of CB in the most common conductive PLA filament from Proto-pasta® is around 21.4 wt% [12, 52].

Preparation of 3D printable filaments from CB-PLA composites is always a compromise between their flowability and electrical properties, which are strictly related to the CB content and suitable dispersion level of the CB in the PLA matrix. Therefore, the electrical properties of the prepared materials were studied by the four point probe method and the results are presented in Fig. 2b. As can be noticed, the resistivity of CB-PLA composites decreased with the higher content. For the CB20 sample, the resistivity was 397±21 Ωcm, while for the CB25 sample, the value of this parameter was 164±30 Ωcm. For comparison, the resistivity for commercial conductive PLA from Proto-pasta® measured with the same technique was 563±41 Ωcm.

FTIR spectroscopy was used to characterise the chemical structure of CB-PLA composites and the results are shown in Fig. 2c. As can be noticed, there are no significant differences between the studied samples. All materials showed characteristic peaks originating from the PLA. The adsorption at 1746 cm^−1^ and 1080 cm^−1^ are related to the presence of C=O groups, the peaks at 2995 cm^−1^ and 2946 cm^−1^ correspond to asymmetric and symmetric vibrations of the –CH_2_– and –CH_3_ groups, while the signals at 1452 cm^−1^ and 1361 cm^−1^ are due to asymmetric and symmetric bending of the CH_2_– and –CH_3_ groups [53]. It was found that the spectra of CB0 and PLA are very similar, which indicates that the PLA degradation during extrusion is rather gentle. In addition, increasing the amount of CB filler in CB-PLA composites does not significantly affect the chemical structure. All FTIR spectra are similar to each other, which indicates that there are no significant surficial changes in the chemical composition of the studied materials.

The thermal stability of the CB-PLA composites was determined by TGA, see Fig. 2d. More detailed data, including derived thermogravimetric analysis (DTG) and individual decomposition temperatures corresponding to the weight loss (*T*_−2%_, *T*_−5%_, *T*_−10%_ and *T*_−50%_), the temperature at which the maximum of the decomposition peaks was reached (*T*_max_) and the char residue remaining at 800°C are summarised in the SI file, Fig. S3a-d and Table S1. For all materials, only one decomposition step was observed, occurring at around 360°C, which corresponds to the thermal decomposition of PLA [54]. The results show that the temperatures associated with the corresponding weight loss (e.g. *T*_-2%_ or *T*_-5%_) are significantly lower for the CB0 sample compared to untreated PLA. This study confirms previous assumptions about the degradation of PLA during extrusion. Furthermore, it can be seen that, as the CB content increased, the temperature corresponding to the maximum weight loss also increased. The addition of the filler improved the heat transfer in the PLA matrix, which resulted in a reduction of the thermal diffusion and thermal decomposition of the PLA matrix. Compared to the CB0, the *T*_max_ parameter for the CB20 material increased by 11.8°C. On the other hand, the CB25 sample was characterised by poorer thermal stability compared to the samples with a lower amount of CB filler. This may be due to the formation of agglomerates and numerous defects in the sample [55], whose presence is suggested by the SEM micrograph in Fig. 2g.

Moreover, it should be highlighted that TGA is a useful tool for determining and verifying the CB filler content in prepared composites. The filler content or final composition of prepared materials for ME 3D printing might be overestimated especially in the case of multi-component materials prepared by simple dosing of all components to the hopper of the relatively low-cost single-screw filament extruders that are easily available on the market. As can be noticed, for the CB-PLA composites with higher amounts of CB filler, the char residue at 800°C also linearly increases, which confirms that the final composition of CB-PLA composites prepared using a laboratory conical twin screw extruder is correct.

Summarising the DSC study, presented in the SI file, section S3, the glass transition temperature, *T*_g_, is comparable for most samples; we only see a decrease in this parameter for the CB25 sample (51.9°C). This observation can be explained by filler aggregation and agglomeration, which resulted in weak matrix-filler interactions [56]. The enthalpy of the crystallisation decreases significantly after processing the material, which is likely caused by the degradation of the polymer chains and hindered chain reorientation [12]. With the addition of CB filler, this parameter increased until the critical CB content was reached at 25% filler. Thus, to a certain extent, CB can act as a nucleating agent for crystallisation [57]. However, too-large amounts resulted in agglomeration of the carbon black, which prevented the movement of macromolecular chains. The *T*_g_ studies determined by DMA and DSC are summarised in SI file, Fig. S3. The reported *T*_*g*_ values of DMA are generally higher than the *T*_*g*_ values measured using DSC [58]. A similar behaviour was observed for the CB-PLA composites. As can be observed, the glass transition temperature measured for the CB25 sample by DSC showed a different trend than for the DMA. This is due to the high content of carbon black (25 wt%). The presence of fillers can enhance the thermal degradation of the polymer matrix during processing [59, 60]. Moreover, partial degradation of the CB25 sample could also occur during the DSC measurement because, compared to the DMA in the DSC, the *T*_g_ was determined after the second heating.

The SEM technique was used to study the surfaces of the conductive CB-PLA composites. The surfaces of the CB20 and CB25 samples are shown in Fig. 2f and g, and the remaining micrographs are included in the SI file, Fig. S4. The dispersion of the filler in the polymer matrix has a significant impact on the morphology of the composites. Depending on the amount of filler, the surfaces of the samples differ from each other. With the increase in the filler content, the surface structure of the sample becomes irregular, and characteristic depressions and protrusions appear, which confirms the agglomeration of the CB particles. A similar relationship was observed by Guo et al. [55].

To sum up, considering melt-flow behaviour, thermal stability and morphology of studied CB-PLA composites, the sample CB20 with 20 wt% of carbon black was chosen as optimal composition for further modification by diamondised nanocarbon powders, and the obtained results and finding are presented in the next sections.

### Physico-chemical characterisation of diamondised nanocarbon powders

**Fig. 3:**
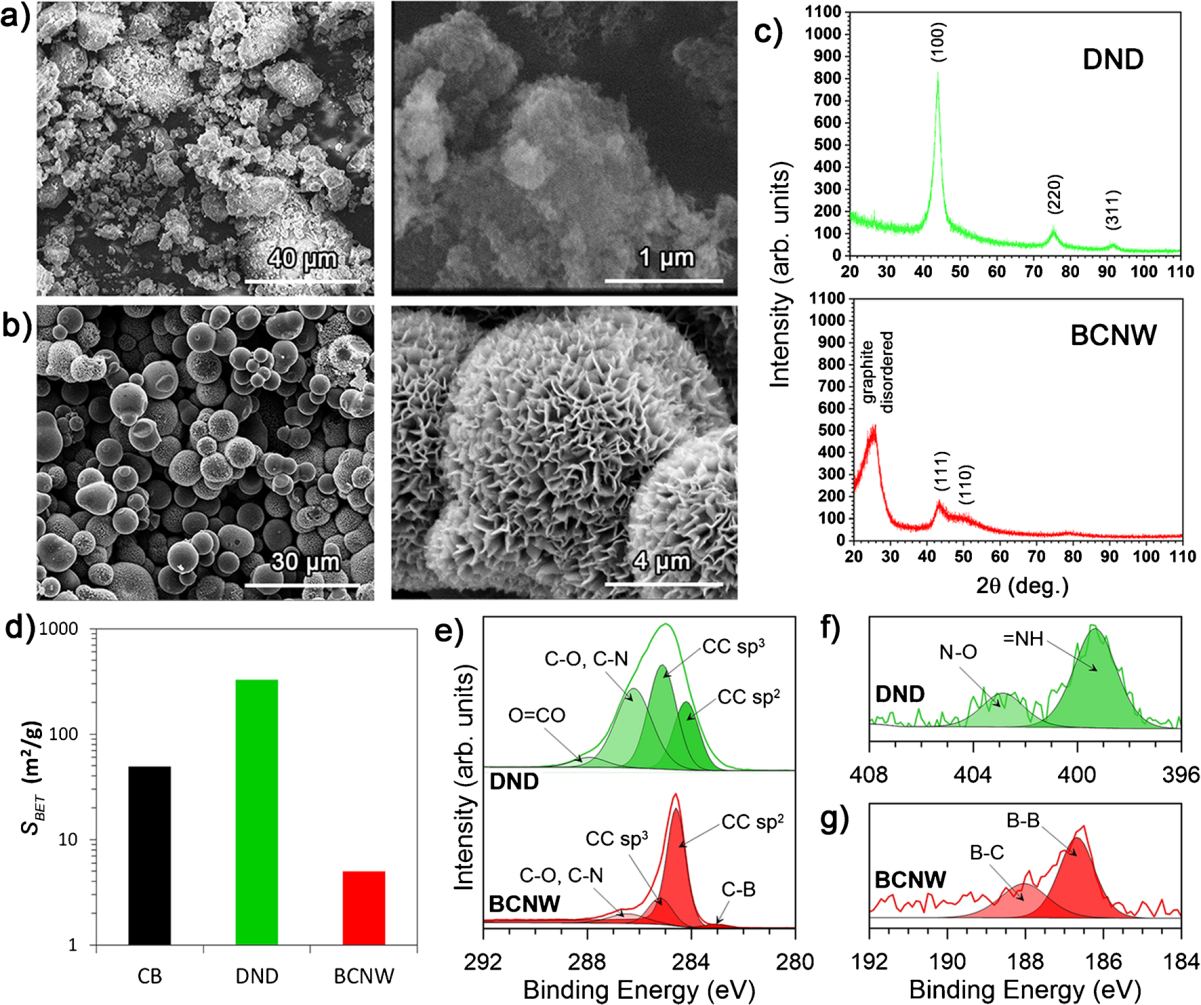
Structural and chemical characterisation of the DNC powders to be used as fillers: (a, b) SEM micrographs for (**a**) DND and (**b**) BCNW grown at GC; (**c**) X-ray diffraction patterns; (**d**) surface area development based on BET isotherm analysis; (e–g) high-resolution X-ray photoelectron spectra for (**e**) *C 1s*, (**f**) *N 1s* and (**g**) *B 1s*

Two different DNC powders were introduced to the conductive PLA-based composite using the procedure explained in detail in the experimental section. DND forms nanometric-sized particles that easily agglomerate into larger structures, as seen in Fig. 3(a), which are predominantly (111)-oriented diamond facets (2θ = 43.9°) with a notable influence of (220) and (311), as revealed by the XRD spectrum in Fig. 3(c). These results correspond well to previously reported XRD analyses [61]. The mean size of the DND crystallites was found 4.4 nm, the value calculated using the Scherrer formula [37].

The micrometre-scale dimensions of the BCNW powders are primarily influenced by the initial size of the glassy carbon spheres that serve as the cores of the core-shell structures, as depicted in Fig. 3(b). The thickness of the BCNW shell layer reaches approx. 100 nm, while its developed structure resembles that obtained for BCNW grown on flat surfaces [41]. The XRD pattern reveals GC-originating peaks (2q~26°), broad due to the disordered character of the graphite layers [62]. The above peaks are overlapped by BCNW-originating (100) graphene facets, with (110) facets also present at approx. 2q = 50.7° [63].

The specific surface area (*S*_*BET*_) was calculated from nitrogen adsorption-desorption isotherms using the Brunauer-Emmett-Teller (BET) equation. It was observed that the BCNW film growth on GC caused an over x3.5 *S*_*BET*_ increase when compared to unmodified GC powder, from 1.5 to 5.2 m^2^/g (see Fig. 3(d)). The *S*_*BET*_ estimated for the DND surpasses the BCNW, as was expected considering the huge disproportion in the average size of both DNC forms, reaching 326 m^2^/g. Such a high value may have a fundamental influence when considering the ease of formation of conductive paths within the PLA-based composite and development of the electrochemical surface area. Comparably, the CB used as the primary PLA filler has an *S*_*BET*_ of 49 m^2^/g. The details of the BET analysis are presented in the SI file, Fig. S5.

The surface chemistry of the DNC powders was also investigated using high-resolution XPS. The spectra recorded at the core-level binding energy of *C 1s* (Fig. 3(e)) reveal the complexity of the detonated nanodiamonds. As expected, the most intense peak at 285.1 eV originated from the *sp*^*3*^-carbon phase forming diamond crystal lattice, while the −0.8 eV shifted component represents graphitic *sp*^*2*^-C. The contribution of these moieties in the total [C] share is 36.6 and 22.0%, respectively, which is similar to other DND studies [64], while the remaining carbon atoms originate from hydroxyl (and imine) and carboxyl bonds. The XPS analysis also confirmed a significant share of nitrogen within DND, 2.9 at.% in total, and originating from pyridinic (399.2 eV) and oxidised pyridinic (402.5 eV) bonds (Fig. 3(f)) [65]. The chemistry of the BCNW films grown on GC is significantly different from DND. The primary *C 1s* peak, which is slightly asymmetric, corresponds to *sp*^*2*^-C (72.2% of the total [C]) while the share of diamond-like sp^3^-C exceeds 15.3% of the total [C]. This BCNW surface chemistry very much resembles the one grown at a flat surface; however, the *sp*^*3*^-C/*sp*^*2*^-C share of 0.21:1 is lower (compared to 0.33:1 [42]), most likely due to not fully developed wall cores, as visible in the SEM images. The boron incorporation is notable in both the *C 1s* as well as *B 1s* (Fig. 3(g)) spectra, where the primary contribution originates from elemental boron (186.7 eV, 0.5 at.%) and B-C interaction (188.0 eV, 0.3 at.%).

Using DND or BCNW as nanofillers can increase the interface between the composite components. The anticipated higher efficiency of DND or BCNW (and spherical nanofillers in general) in improving the mechanical properties of nanocomposites is due to a shape/geometry of diamondised particles, interphase volume and interfacial area per unit of the nanoparticle volume. Furthermore, DND poses superior surface chemistry due to its rich, *sp*^*3*^-carbon hybridisation, which allows for a greater variety of possible chemistries and a higher number of surface functional groups per particle [66].

### Role of diamondised nanocarbon fillers on thermo-mechanical and structural properties of studied composites

**Fig. 4:**
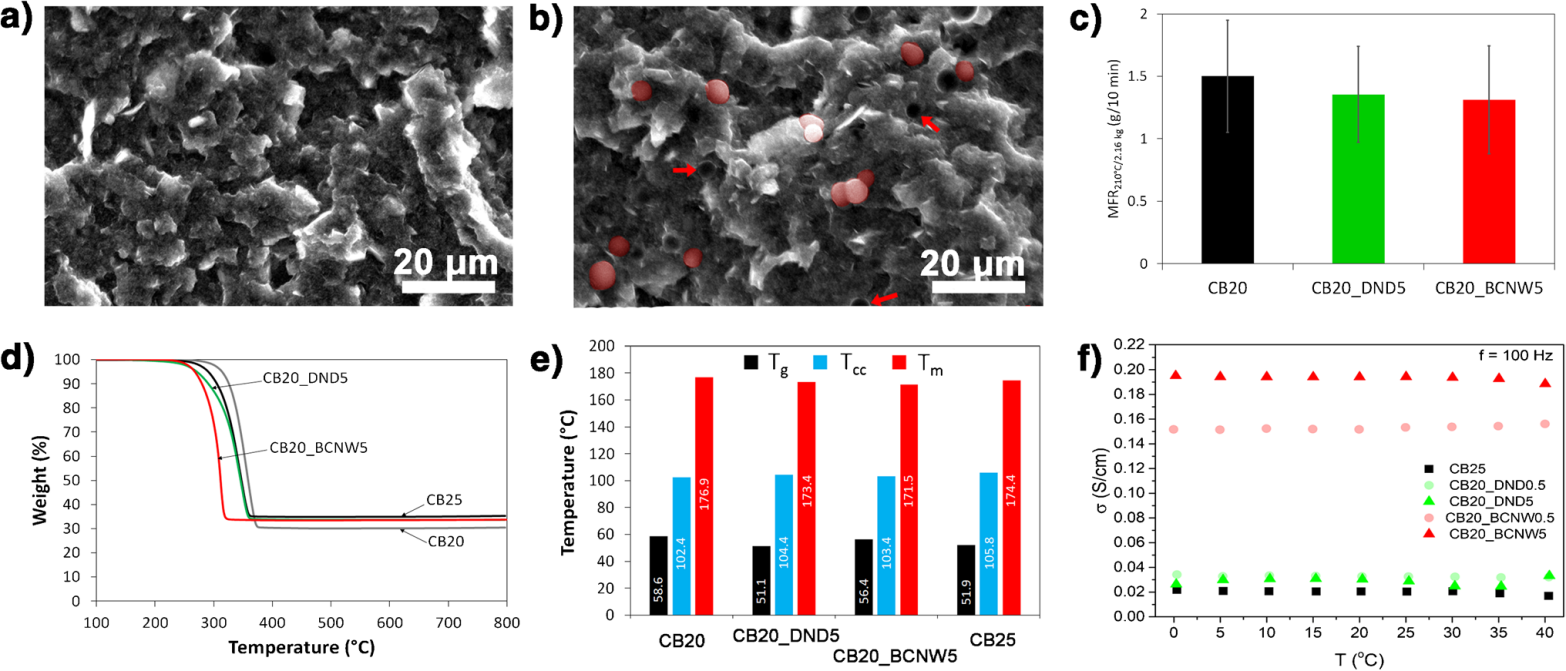
Structural and performance properties of CB-DNC-PLA composites with DNCs (DND, BCNW): **a**, **b** SEM micrographs for **a** CB20_DND5 and **b** CB20_BCNW5; **c** MFR; **d** TGA; **e** DSC; **f** BDS conductivity analyses

Figure 4a shows the surface morphology of PLA-based composites with CB and DND fillers (CB20_DND5). As a powder, DND tends to form agglomerates, as shown in Fig. 4a. The method of preparing polymer composites breaks up agglomerates and leads to a homogeneous distribution of DND throughout the volume of the material; however, individual DND crystallites are not visible due to their nanometric size. In the case of CB20_BCNW5 (Fig. 4b), uniformly distributed BCNW at GC particles (highlighted in red) are visible on the surface of the composite. At the same time, the red arrows mark the voids left by the BCNW at GC that were detached from the surface during filament breakdown, indicating low adhesion forces to the PLA matrix. It is assumed that the BCNW films are partially delaminating from the GC surface during thermal mixing of the composite, an observation made considering the semi-bare GC surface under the microscope in Fig. 4b and, at a higher magnification, in the SI file, Fig. S6.

The rheological properties of the CB-DNC-PLA composites were determined by MFR measurement and the obtained results are shown in Fig. 4c. As can be observed for the CB20_DND5 and CB20_BCNW5 samples, the MFR_210°C/2.16kg_ were similar, in the range of 1.31–1.36 g/10 min, which is 10–15% lower compared to CB20 without DNC fillers. This indicates that DNCs decrease the PLA mobility, which results in the increasing viscosity of CB-DNC-PLA composites. However, the viscosity of the CB-DNC-PLA is not as high as could be expected. As presented in Fig. 2a, for the CB25 sample, characterised by the same total filler content (25 wt%), it was impossible to measure the MFR at 210°C and load of 2.16 kg. Furthermore, Sahu and Sreekanth [67] recently published interesting studies about the effect of DNCs, CNT and graphite nanoplatelets on the mechanical, thermal and rheological properties of high-density polyethylene. The melt flow rate results showed that regardless of the type of carbonic nanofillers used, 0.1 wt% of nanofiller in polyethylene results in a decreased MFR. However, the highest viscosity (lowest MFR) was observed for composites with diamondised nanoparticles, for which the MFR decreased by 38% compared to pure polymer. Surprisingly, the MFR results for studied CB-DNC-PLA composites showed completely different tendency compared to findings described by Sahu and Sreekanth [67] and the addition of DNC (5 wt%) had negligible impact on MFR parameter. This indicates that the combination of CB and DNCs at high concentrations could enhance the PLA matrix degradation during melt-compounding of CB-DNC-PLA.

To verify this assumption, a thermal stability assessment of the CB-DNC-PLA composites was performed and the results of the TGA and DTG are shown in Fig. 4d and SI file, Fig. S3d, respectively. It is seen that the presence of DNCs, regardless of their type, results in a decrease in the decomposition temperature of the composite. This observation is surprising, especially considering that DNCs are characterised by relatively high thermal stability and usually improve the thermal properties of polymer composites [68, 69], which is related to the presence of surface termination of oxidised functional groups, such as –OH, –C=O, –COOH and –C–O–, usually delaying the initiation phase during thermal degradation. Zhao et al. [70] investigated the effect of DNCs (used in the range of 0.1–5 wt%) on the structure and properties of PLA. TGA results clearly show that DNCs improved the thermal stability of the composites. Similar findings were also described by Shi et al. [71], who studied PLA composites reinforced by diamondised nanocarbon modified by polyethylene glycol (nanofillers used in the range of 0.5–2 wt%). On the other hand, Bikiaris [72] in a comprehensive review work focused on answering the question *Can nanoparticles really enhance thermal stability of polymers?* which indicates that selected nanofillers can accelerate the decomposition of PLA, which is related to the aggregation and agglomeration of nanofillers and therefore the formulation of a microcomposite rather than a nanocomposite. Indeed, Barcelos et al. [73] studied the thermal stability of poly(3-hydroxybutyrate-co-3-hydroxyvalerate) reinforced by diamondised nanocarbons (10 wt%). The results showed that the onset degradation temperature was 8% lower than that for the pure polymer. The authors indicated that this phenomenon is related to interactions between the PLA and DNC phases. According to Chen et al. [74], well-distributed nano-sized hydroxyapatite (used in the range of ~9–33 wt%) can catalyse polymer decomposition and obstruct the degradation by-products from diffusing out of the composite material. It should be emphasised that a relatively high content of fillers was applied in this study, and therefore, the probability of agglomeration and aggregation of those fillers is also high. Moreover, as can be noticed, the sample with BCNW was more prone to thermal decomposition compared to the sample with DND, which is related to the dispersion level of the used fillers, and as a consequence, the matrix-filler interactions, as confirmed by SEM analyses (see Fig. 4a, b).

The effect of the diamondised nanocarbons on the thermal behaviour of the CB-DNC-PLA composites determined by DSC is shown in Fig. 4e. It can be seen that the type of DNC nanofillers added affects the glass transition temperature of the PLA matrix. In the case of the materials containing DND, the obtained *T*_g_ result was lower compared to BCNW. In addition, the glass transition temperature of the sample with DND was lower than that determined for CB25. This is due to the improved dispersion of BCNW in the PLA matrix, compared to DND (see Fig. 4a, b) and, as a consequence, enhanced supramolecular matrix-filler interactions [75], and so an increase of *T*_g_ [76]. The cold crystallisation temperature increases when DNCs are added. Increasing the amount of additives in the material creates difficulties in its ordering, so an increase in temperature is required to transform the material into a more crystalline structure. However, it can be noted that BCNW has a lower *T*_cc_ and *T*_m_ compared to DND. This also indicates the improved dispersion of BCNW in the PLA matrix, which could limit the formation of matrix-filler structure impairing crystallisation due much lower thermal conductivity [77].

The auxiliary infrared and Raman spectroscopy analyses are presented in SI file, section S7. The addition of any diamondised nanoparticles does not affect the chemical structure of PLA. Raman spectra patterns are dominated by CB-originated bands [78], which are overlapping both the DND and BCNW signals. The D/G ratios of the CB-DNC-PLA composites (CB20_BCNW5 and CB20_DND5) are slightly lower compared to PLA with only CB, as the G band in both DND and BCNW is strong and narrow, while the D band signals are weakened after mechanical treatment [79].

The electrical parameters of the CB-DNC-PLA samples were studied by BDS. All samples showed AC conductivity independent of frequency. Figure 4f presents the AC conductivity versus the measured temperature for the composites containing DND and BCNW (0.5 and 5 wt%) and the comparative CB20 sample. The lack of thermal activation indicates an electronic conduction mechanism assigned to nanofiller carbon assembled in the percolation paths. The conductivity values varied from 0.01 to 0.2 S/cm. It can be seen that the CB20 exhibits the lowest conductivity, yet no distinctive changes are visible for the sample containing DND, even at its highest concentration. The possible explanation comes from the origin of DND’s conductivity, related to the presence of structural defects, surface termination [80] but also water adsorption [81]. The lack of bulk conductivity results in a negligible effect of the DND on the conductive paths within the CB-DND-PLA composite. However, one order of magnitude higher conductivity is observed after BCNW-loading, which increases with the BCNW content increase. The composite reveals initially unexpected behaviour, since BCNW particles dimensions are too large to contribute to the formation of conductive paths. However, the SEM micrographs in Fig. 4b and in SI file, Fig. S6 provide a plausible explanation. A flat GC surface indicates that the BCNW film was detached from it during filament processing and was dispersed within the polymer matrix. This observation corroborates the electrical conductivity increase of the CB20_BCNW5 composite.

The reason for the observed enhancements can be credited to beneficial interactions between oxygen-containing groups (such as COOH, C=O and similar) that are present on the surface of DND, and the OH groups of PLA [66]. It is possible that by heating, weakly bonded species such as adsorbed hydrocarbons are removed from the surface of DND or BCNW, which facilitates weak interactions, mainly hydrogen bonding, between these groups and the functional groups of the polymer matrix. Collectively, these interactions lead to a stronger nanofiller-matrix interface that increases polymer crystallinity [82]. PLA exhibits nanoparticle attractive interactions by replacing the DND’s polar functional groups that are prone to hydrogen bonding and other stronger interactions with alkyl chains, which can only interact via weak van der Waals forces [83]. Additionally, the improvement in mechanical properties of PLA due to DND-PLA can be attributed to favourable interactions and entanglement between DND grafted PLA chains and the matrix [84].

### Electrocatalytic properties of diamondised nanocarbon-filled PLA composites

The electrocatalytic properties of the 3D-printed conductive composite electrodes were evaluated using a popular redox species, potassium ferrocyanides [Fe(CN)_6_]^3−/4−^, after initial surface activation to uncover conductive carbon (CB and DNC) fillers. Despite its common use, one must consider that this redox probe is characterised by an inner sphere electron transfer (ISET) mechanism, which may significantly hinder the redox process depending on the surface chemistry, i.e. the presence of oxidised species terminating the electrode surface [48].

**Fig. 5:**
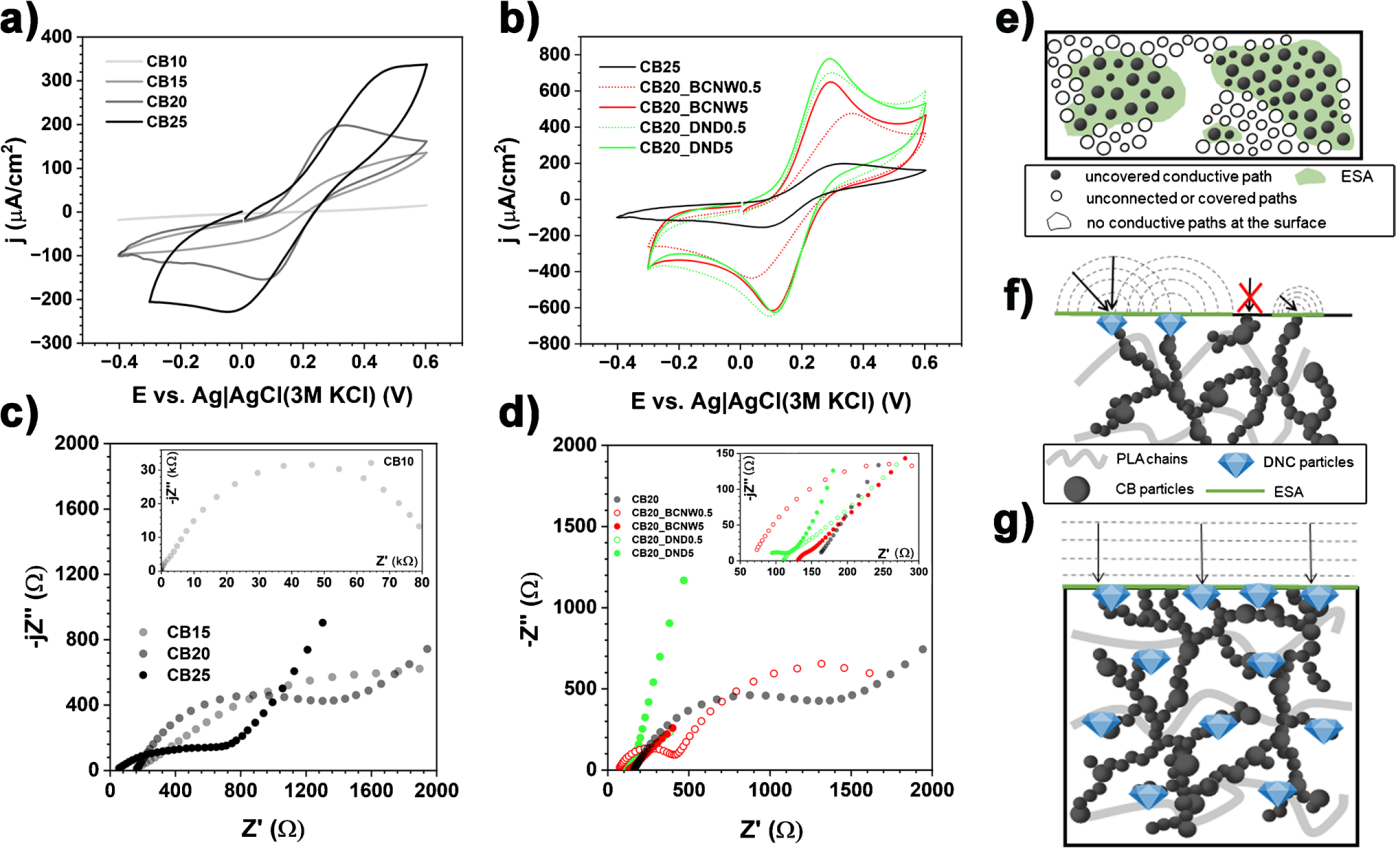
Electrochemical studies of 1 mM [Fe(CN)_6_]^3−/4−^ oxidation/reduction in 0.1 M KCl processes at the surface of **a**, **c** CB-PLA and **b**, **d** CB-ND-PLA composites: **a**, **b** CV at a scan rate of 100 mV/s; **c**, **d** EIS analyses; **e**–**g** schematic visualisations: **e** top view, electrode surface heterogeneity dependent on filler distribution and activation; **f** side view, charge transfer kinetics of different electrolyte/filler particles; **g** side view, electrode homogeneity for heavily overlapping diffusion layers with multiple percolation paths

Since conductivity is obtained in the composite via carbon black percolation paths, the influence of the concentration of CB is illustrated in Fig. 5a and c. The cyclic voltammetry analysis reveals the linear I-V characteristics below 15 wt% of the carbon black filler, which is expected considering the very high resistivity of the CB10 sample (Fig. 2b). The higher the number of conductive paths, the lower the resistance overpotential of the electrochemical reaction and the more thermodynamically favoured the redox event. In the case of CB15, the fractional surface coverage by uncovered conductive paths was too small to record redox peaks in the studied polarisation range [11]. The minimum amount of the used CB filler to ensure effective charge transfer through the electrode/electrolyte interphase was 20 wt% (CB20), producing [Fe(CN)_6_]^3−/4−^ redox peak separation Δ*E* = 250 mV, measured at a 100 mV/s scan rate. Together with a slightly unbalanced anodic-to-cathodic current ratio *i*_p,A_:*i*_p,C_ = 1.30, it hints at irreversibility of the studied process. Notably, the electrocatalytic efficiency will further depend on the type of studied CB (i.e. surface area development or termination groups), which was not studied here. Moreover, it should be considered that an insufficient amount of conductive paths will electrically cut off a part of the electrode surface, as illustrated schematically in Fig. 5e and f. For a random distribution of electric heterogeneity areas, a specific type of partially blocked electrode surface kinetics takes place [11, 36]. One should further consider a similar event in the case of ineffective surface activation [85], as illustrated by the blank circles in Fig. 5e.

Semi-overlapping adjacent diffusional fields are reflected in the vestigial voltammetry characteristics by the microelectrode. According to Davies et al. [36], the effect will result in smaller cathodic peak currents and large diffusional tails, a feature seen in the CB20 and CB25 samples. If these insulating parts of the electrode are sufficiently small, though, the adjacent electro-active surface areas (ESA) start to overlap. The studied redox process improved upon a CB content increase. A similar conclusion was drawn for the CB-PLA composite fabricated by Stefano et al. [86], who report 28.5 wt% of CB. With a sufficient number of conductive paths, the heavily overlapping diffusion fields act similarly to planar diffusion to a homogeneous surface (Fig. 5g). Interestingly, the CVs for CB25 again resulted in a Δ*E* increase of up to 523 mV and *i*_p,A_:*i*_p,C_ = 1.45, suggesting mechanistic complications in the electrode process; see the SI file, Table S4, for details. A plausible explanation of the reversibility decrease, suggested by the altered sample topography (SI file, Fig. S4d), is the agglomeration of the excess CB particles breaking some available percolation paths. The formation of secondary CB aggregates and rearrangement of percolation paths may lead to deterioration of the electrocatalytic properties [12]. Depending on the source and chemistry of fillers, their agglomeration tendency may vary.

The impedimetric studies corroborate the above findings (Fig. 5c); see the SI file, Table S5, for a detailed interpretation. The higher the carbon black content, the lower the charge transfer resistance, *R*_CT_. In particular, for the CB15 and CB20 samples, flattened semicircles can be recognised, characteristic of frequency dispersion of capacitance, whose surface distribution is an effect of the spatially heterogeneous electric properties. The lowest *R*_CT_ value was found for the CB20 (1130 Ω) and CB25 (630 Ω) samples, yet, in the latter, the frequency dispersion of the capacitance increased again. The impedance spectra of both these samples possess a linear segment in the mid-to-low frequency range, which should be interpreted as Warburg diffusion impedance, a sign of a diffusion-controlled process at low relaxation times.

As noted before, the CB20 sample was taken as the basis for the DNC additions, which results from the thermo-mechanical properties of the studied composites and the electrocatalytic efficiency of the 3D-printed electrodes, as testified by the CV and EIS analyses. The addition of 5 wt% of any of the studied DNC fillers resulted in a significant enhancement of the redox process peak currents *i*_p,A_ and *i*_p,C_. This effect is caused by lower redox activation overpotentials at the DNC surface compared to CB, which originate from the lower adsorption energy of [Fe(CN)_6_]^3−/4−^, and, as a consequence, the ease of electron transfer by this ISET probe. This, in turn, drives the increase in the heterogeneous rate constant *k*_0_. The relationship is represented as Eq. (1) for the irreversible one-step, one-electron transfer process [87]:

1 $$i= FA{C}_O^{\ast }{D}_O^{1/2}{\nu}^{1/2}{\left(\frac{\alpha F}{RT}\right)}^{1/2}{\pi}^{1/2}\chi$$

where *A* is the surface area, *T* is the temperature, *C*_O_* is the bulk concentration and *D*_O_ is the diffusion coefficient of the oxidised species, *υ* is the scan rate, α is the transfer coefficient, and *F* and *R* are the Faraday and gas constants, respectively. Here, *χ* is the kinetic parameter representing the current function for the irreversible charge transfer dependent on *k*_0_. When the diffusion layers are not overlapping, an additional, spherical correction factor ϕ should be considered, and it should include the individual ESA radius *r*_0_, Eq. (2):

2 $$i={i}_{(plane)}+\frac{FA{D}_O{C}_O^{\ast}\phi }{r_0}$$

Both the $\chi ={\chi}_{\left({k}_0\right)}$ and $\phi ={\phi}_{\left({k}_0\right)}$ functions were introduced in [87], and increase as *k*_0_ increases. The *i*_p,A_ value reaches 259 μA for the CB20_BCNW5 and 312 μA for CB20_DND5 sample, nearly 4× higher compared to original the CB20 and 2.5× higher than CB25 having the same total wt% of all conductive carbon fillers. It is plausible that, due to the different CB-PLA and CB-DNC-PLA interactions, the effect may be enhanced by more effective PLA removal during surface activation. The electrode kinetics enhancement is also represented by a more uniform *i*_p,A_:*i*_p,C_ ratio, which is equal to 1.27 and 1.07 for CB20_DND5 and CB20_BCNW5, respectively. Last but not least, a notable drop in Δ*E* is observed in both cases, down to 170 (BCNW) and 192 mV (DND), testifying a more reversible charge transfer mechanism; see details in the SI File, Table S4. Moreover, incorporation of DND and BCNW partially saturated by COOH mediating electron transfer because of faster electron transport via network of DNC and CB [88].

The approximate distance, δ, diffused by the redox species is given by Eq. (3).

3 $$\delta ={\left(2D\frac{\Delta E}{\nu}\right)}^{1/2}$$

for [Fe(CN)_6_]^3−^, the value *D*_O_ = 6.67×10^−6^ cm^2^/s [17], which for the used scan rate (ν = 100 mV/s), estimates the diffused distance between 50 and 70 μm. The SEM micrograph in Fig. 4b reveals that BCNW particles are loosely scattered in the polymer matrix. Despite the evident removal of certain BCNW particles during sample preparation, a portion of the particles remained intact. Thus, the value of δ is sufficiently large to enhance the electrocatalytic properties when 5 wt% of BCNW was added. However, at lower BCNW admixtures, the EHE effect is not provided effectively. The Δ*E* drops to 310 mV, *i*_p,A_ drops to 190 μA and even the *i*_p,A_:*i*_p,C_ symmetry is diminished to 1.15. Such a situation is not observed in the case of the DND admixture, since the much smaller DND particles are evenly scattered in the polymer matrix even at lower quantities and the heavily overlapping diffusion fields (Fig. 5g) remain even when only 0.5 wt% of DND is used. The Δ*E* increases merely by 10 mV, while the peak current symmetry remains intact (*i*_p,A_:*i*_p,C_ = 1.1).

Finally, the EIS results for these samples are presented in Fig. 5d and generally confirm the CV analysis. Troublesome enough, there is no electric equivalent circuit (EEC) universal enough to fit the impedimetric data of all the studied samples. The reason behind this is the different reversibility of the electrochemical process at each electrode, influencing the appearance of diffusion Warburg impedance or a second time constant due to the different relaxation times of the electron transfer through different types of conductive carbon fillers-electrolyte interfaces in several cases. Following the use of different EECs, the comparative accuracy is limited. The EEC details are as presented in the SI file, Table S5. However, regardless of the studied composite, the *R*_CT_ drop is observed following the ND addition, reaching as low as ~30 and 50 Ω for the CB20_BCNW5 and CB20_DND5 samples, respectively, down from ~1100 Ω for CB20. The investigated CB-DNC-PLA composite electrodes hold promise in the development of high-performance electrochemical devices, such as sensors and supercapacitors, owing to their superior electrical conductivity and mechanical properties. The use of 3D printing technology in their fabrication offers advantages in terms of cost-effectiveness, design flexibility and ease of customisation [85].

**Fig. 6:**
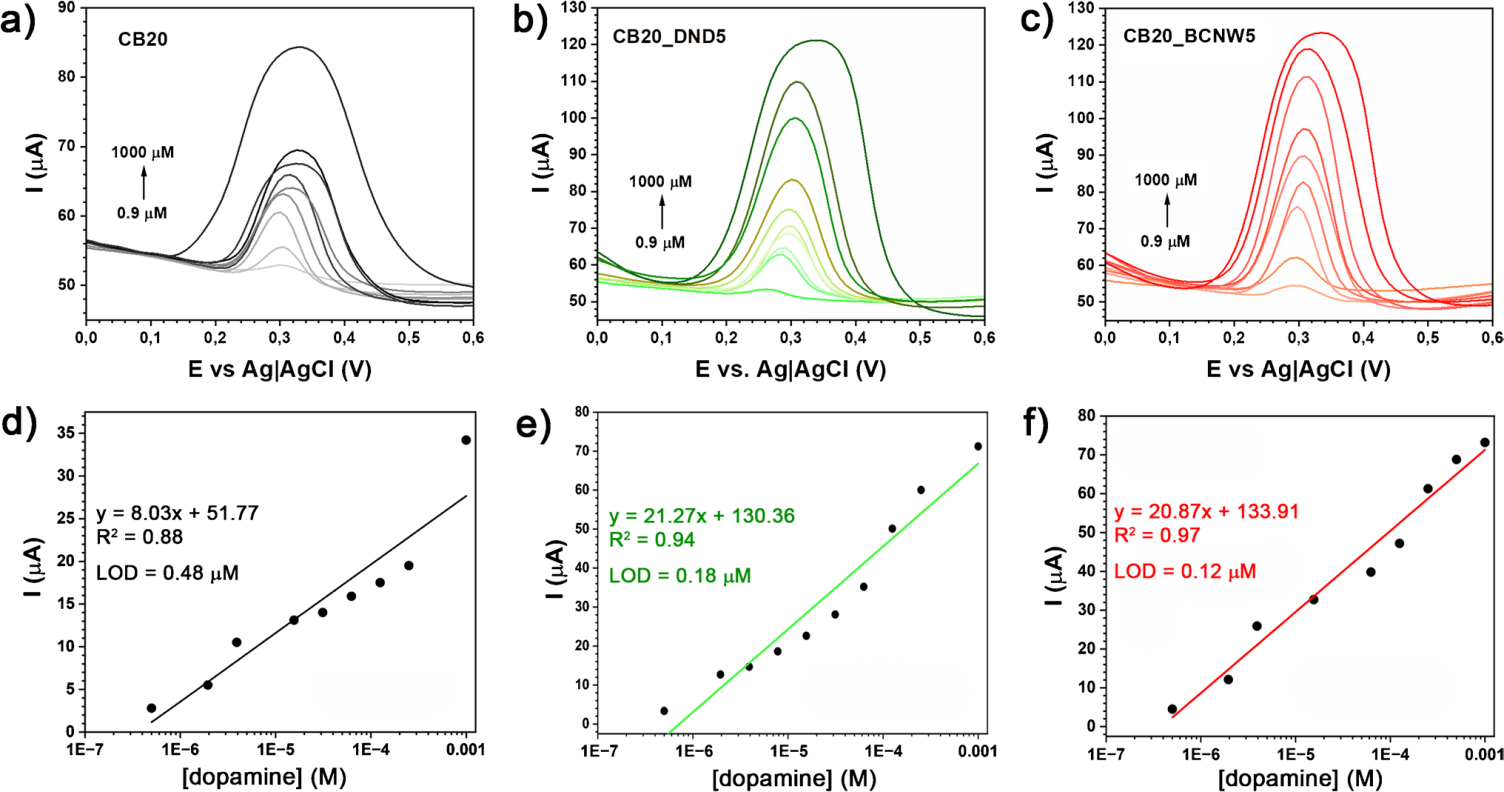
The DPV scans for dopamine detection in 0.05 M PBS (pH = 4.5) with corresponding calibration curves, using different 3D-printed electrodes: **a**, **d** CB20; **b**, **e** CB20_DND5; **c**, **f** CB20_BCNW5

Finally, the 3D-printed composite electrodes, namely CB20 (Fig. 6a, d) CB20_DND5 (Fig. 6b, e), and CB20_BCNW5 (Fig. 6c, f), were evaluated for electrochemical detection of dopamine in PBS solution (pH = 4.5). Dopamine was selected due to its critical role as a neurotransmitter and ease of results comparability with other sensors relying on electrochemical detection [89]. These results were obtained for increasing dopamine concentrations after the electrochemical activation procedure.

The LOD was estimated with LOD = 3 × RSD/slope, where RSD is the relative standard deviation in the low concentration range [90]. The obtained data reveal a considerate improvement of analytical characteristics upon DNC addition to the CB-PLA electrode, where the lowest LOD (0.12 μM) was obtained for the CB20_BCNW5 electrode. The obtained LOD values reveal the high potential of fabricated composites for electroanalytic applications, being competitive with the data obtained in analogous experiments on 3D-printed CB-PLA electrochemical platforms [91, 92]. Interestingly, the increase in calibration curve slope (2.6×) and in particular LOD (4.0×) compared to CB20 (LOD = 0.48 μM) is similar to previously observed [Fe(CN)_6_]^3-/4-^ redox kinetics improvement. This effect should be associated with excellent electric conductivity and the electrocatalytic effect of the DNCs [93], which lowers the activation energy barriers of redox processes, increasing the heterogeneous rate transfer, and improving the electrochemical signal response. One should expect that incorporating DNCs into different filaments will analogously improve their electrocatalytic properties. The calibration curve linearity coefficient *R*^2^ was also recognised as higher in the presence of DNCs, a feature possibly related to their wettability by PLA.

## Conclusions

Mixtures of CB and diamondised nanocarbons were studied as fillers for a new PLA-based 3D printable composite. The high electric conductivity was assured by the carbon black filler. CB also reduces the mobility of polymer chains, increasing the composite viscosity. The loading of 20 wt% was chosen as optimal, and its higher content may lead to filler agglomeration within the polymer matrix and deterioration of thermo-mechanical properties (percolation occurs above 15 wt% CB).

Two types of diamondised nanocarbons were used in this study, namely, detonation nanodiamonds (DNDs) and boron-doped carbon nanowall films grown at micrometre-sized glassy carbon powders (BCNWs). Low DNC concentration (up to 5 wt%) was sufficient to increase the composite electrocatalytic performance, confirmed by CV and EIS studies. DNC-containing composites had significantly increased [Fe(CN)_6_]^3−/4−^ reversibility, manifested by a Δ*E* decrease from 300 mV (for CB20) to 191 and 172 mV, for CB20_BCNW5 and CB20_DND5, respectively. The nearly four times higher anodic peak currents also demonstrate a heterogeneous rate constant increase after DNC loading. For the DND filler, the effect was observed even at a much lower load of 0.5 wt%. Our studies also revealed the catalytic effect of DNCs in 3D-printed electrodes, manifested by considerably higher dopamine LOD during DPV studies (0.12 μM for BCNW, 0.18 μM for DND), compared to CB-PLA (0.48 μM).

The results showed that PLA degradation occurs during thermo-mechanical processing via extrusion, but at the same time carbon black filler can stabilise PLA processing. Undoubtedly, diamondised nanocarbons enhanced the thermal degradation of the studied materials, which was confirmed by thermogravimetric analysis and melt flow rate measurements. The FTIR and Raman spectroscopy results confirmed that the chemical structure of the studied composites is similar within the studied range of CB and DNC fillers, not interacting chemically with the PLA matrix.

A conclusion may be drawn that DNCs enhance the electrocatalytic properties of the composite but lead to its thermo-mechanical degradation. The influence of DNCs on the plasticising system of the extruder must also be carefully studied. Therefore, a balance between the rheology and conductivity has to be sought. In small amounts, diamondised nanocarbons are proven to reduce filler agglomeration within the polymer matrix, while sufficient to improve the electrochemical response, making this type of material a worthy candidate for electroanalytic applications.

## Supporting information

ESM 1 The file contains detailed thermal decomposition information, SEM topographies for all the fabricated filaments and GC surfaces in the CB-BCNW-PLA composites, supplementary BET analyses and Raman spectroscopy information, a visualisation of the electrochemical setup, details on the electrochemical activation, and on the CV and EIS analyses together with the proposed EEC fitting results. (DOCX 4449 kb)
