# Supplementary material for: Tailoring diamondised nanocarbon-loaded poly(lactic acid) composites for highly electroactive surfaces: extrusion and characterisation of filaments for improved 3D-printed surfaces
Source: Mikrochim Acta. 2023 Aug 28;190(9):370. doi: 10.1007/s00604-023-05940-7 (PMC10462739; doi:10.1007/s00604-023-05940-7)
Supplement: ESM 1 — The file contains detailed thermal decomposition information, SEM topographies for all the fabricated filaments and GC surfaces in the CB-BCNW-PLA composites, supplementary BET analyses and Raman spectroscopy information, a visualisation of the electrochemical setup, details on the electrochemical activation, and on the CV and EIS analyses together with the proposed EEC fitting results. (DOCX 4449 kb) [file 604_2023_5940_MOESM1_ESM.docx]

Supplementary Information file

For

**Tailoring Diamondised Nanocarbon-Loaded Poly(lactic acid) Composites for Highly Electroactive Surfaces: Extrusion and Characterisation of filaments for Improved 3D-printed Surfaces**

M. Cieślik^1,2,*^, A. Susik^3^, M. Banasiak^4^, R. Bogdanowicz^4^, K. Formela^3^, J. Ryl^2,*^

## ^1^Department of Analytic Chemistry, University of Gdańsk, Wita Stwosza 63, 80-308 Gdańsk, Poland

## ^2^Division of Electrochemistry and Surface Physical Chemistry, Faculty of Applied Physics and Mathematics, Gdańsk University of Technology, Narutowicza 11/12, Gdańsk, 80-233, Poland

## ^3^Department of Polymer Technology, Faculty of Chemistry, Gdańsk University of Technology, Narutowicza 11/12, 80-233 Gdańsk, Poland

## ^4^Department of Metrology and Optoelectronics, Gdańsk University of Technology, Narutowicza 11/12, 80-233 Gdańsk, Poland

^*^ corresponding authors: [mateusz.cieslik@ug.edu.pl](mailto:mateusz.cieslik@ug.edu.pl) (M.C), [jacek.ryl@pg.edu.pl](mailto:jacek.ryl@pg.edu.pl) (J.R.)

**S1. Working electrode and the electrochemical setup**

**
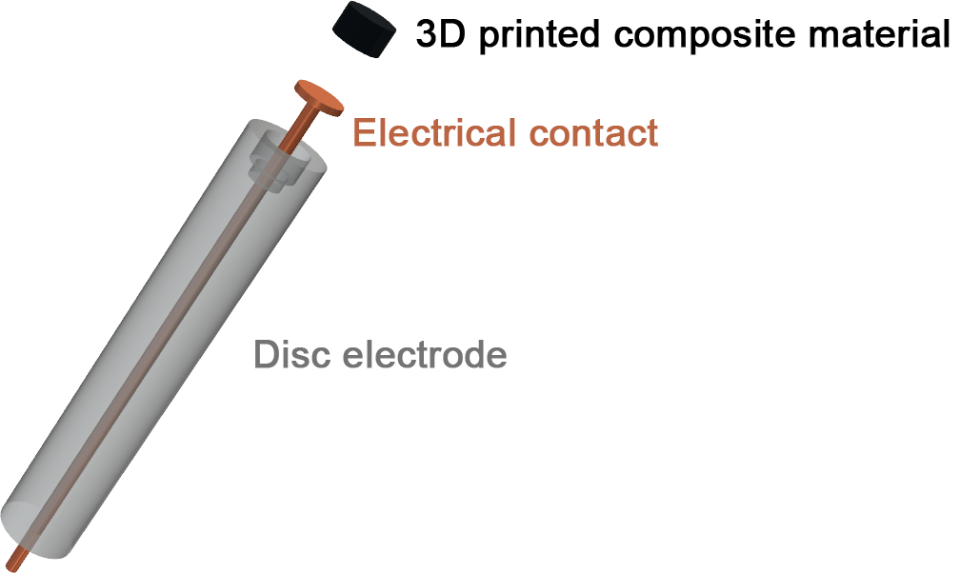
**
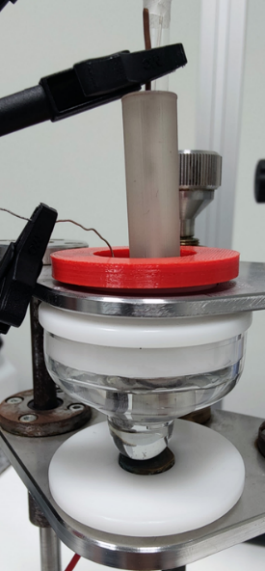


**Figure S1** – Electrochemical setup for 3D printed disc electrode with fabricated composite material printed using a 3D Pen.

**S2. The activation of the CB-PLA and CB-ND-PLA samples by cyclic voltammetry in 1M NaOH**

The electrochemical activation was carried out by potentiodynamic polarization at a constant scan rate of 100 mV/s in a potential range from -1.4 to 1.2 V in 1M NaOH solution. Each electrode was subjected to 10 polarization cycles. Electrode setup: working electrode – composite electrode, reference electrode – Ag|AgCl (3M KCl), counter electrode – platinum mesh.

**
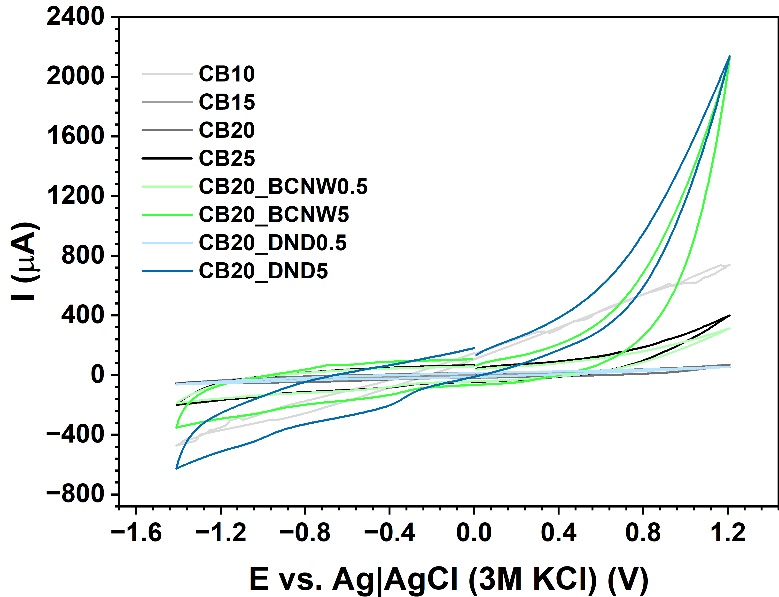
**

**Figure S2** – CV scans registered during electrochemical activation in 1M NaOH.

**S3. Detailed thermal decomposition studies of CB-PLA and CB-DNC-PLA composites**

*
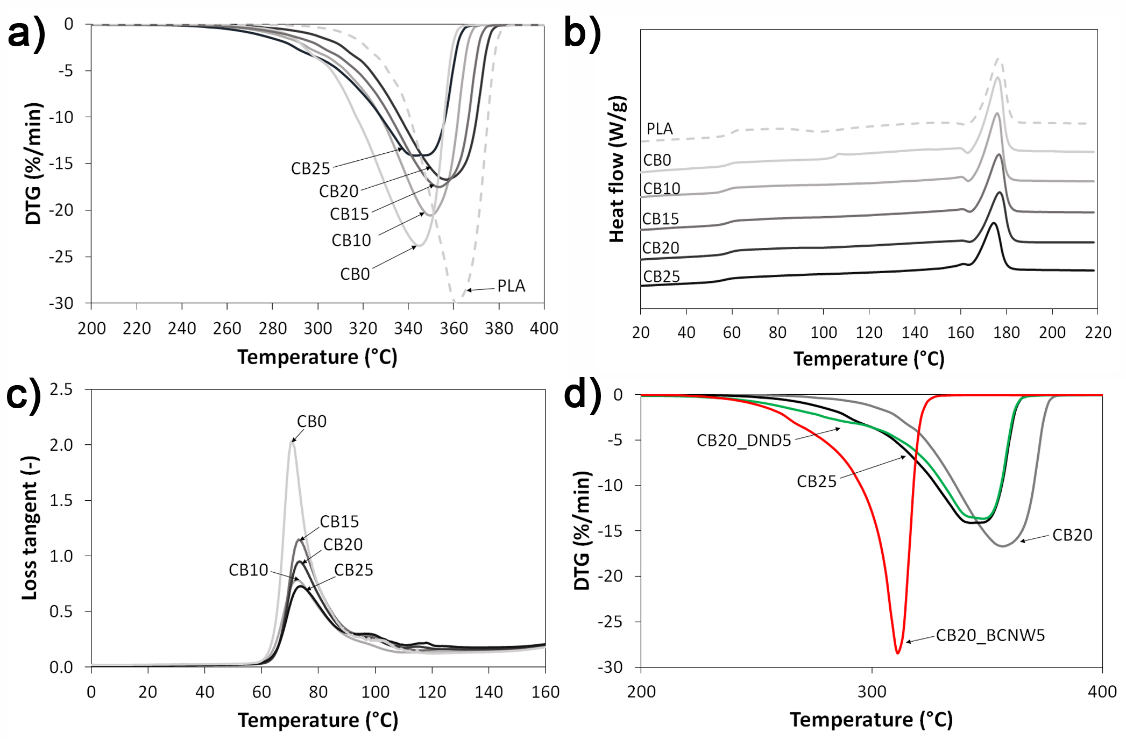
*

**Figure S3** – Processing, thermal and structural properties of CB-PLA composites with different CB contents: a) DTG f) heat flow, g) loss tangent; d) DTG for CB-DNC-PLA.

**Table S1** – Thermal decomposition temperatures and residue mass of PLA_CB and PLA_CB_ND composites

| **Sample code** | **Temperature decomposition (°C)** | | | | **T_max_ (°C)** | **Residue Mass**  **at 800°C** |
| --- | --- | --- | --- | --- | --- | --- |
|  | **T_-2%_** | **T_-5%_** | **T_-10%_** | **T_-50%_** |  |  |
| PLA | 318.2 | 328.7 | 359.4 | 359.4 | 361.0 | 11.3 |
| CB0 | 290.5 | 301.0 | 310.9 | 338.4 | 344.8 | 11.3 |
| CB10 | 283.3 | 298.6 | 312.0 | 346.4 | 349.6 | 20.8 |
| CB15 | 287.4 | 303.8 | 317.2 | 352.5 | 353.5 | 25.8 |
| CB20 | 298.0 | 314.1 | 325.8 | 358.6 | 356.6 | 30.5 |
| CB25 | 274.1 | 291.2 | 305.7 | 347.3 | 342.8 | 35.4 |
| CB20_DND5 | 246.9 | 270.9 | 290.8 | 346.0 | 348.1 | 33.7 |
| CB20_BCNW5 | 253.3 | 267.3 | 279.5 | 311.3 | 311.5 | 33.8 |

The thermal properties of the tested materials were also characterised using differential scanning calorimetry (DSC). The results obtained during the measurements were based on the heat released or absorbed by the material during the test. DSC plots obtained for the PLA/CB materials are shown in **Fig. S3b**, while more detailed data such as the glass transition temperature (T_g_), crystallisation temperature (T_cc_), melting temperature (T_m_), crystallisation enthalpy (∆H_cc_), and melting enthalpy (∆H_m_) are summarised in the **SI file, Table S2**.

**Table S2** – DSC results for PLA_CB and PLA_CB_ND composites

| **Sample code** | **T_g_ (°C)** | **T_cc_ (°C)** | **ΔH_cc_ (J/g)** | **T_m_ (°C)** | **ΔH_m_ (J/g)** |
| --- | --- | --- | --- | --- | --- |
| PLA | 60.0 | 100.2 | -19.7 | 176.7 | 31.0 |
| CB0 | 58.9 | 104.8 | -26.6 | 176.2 | 30.4 |
| CB10 | 57.2 | 105.0 | -25.6 | 175.9 | 27.2 |
| CB15 | 58.2 | 104.1 | -22.6 | 176.8 | 25.1 |
| CB20 | 58.6 | 102.4 | -19.4 | 176.9 | 22.8 |
| CB25 | 51.9 | 105.8 | -21.3 | 174.4 | 22.1 |
| CB20_DND5 | 51.1 | 104.4 | -21.5 | 173.4 | 19.6 |
| CB20_BCNW5 | 56.4 | 103.4 | -21.5 | 171.5 | 20.8 |

The melting point was similar for all samples, while the heat of the fusion decreases as the amount of filler increases. This could mean that CB influences the crystallisation process and contributes to the formation of thinner PLA crystals, which require less heat for the phase transition [1].

The results of the loss tangent as a function of temperature determined for the CB-PLA composites are presented in **Figure S3c**. It was found that the glass transition of the PLA slightly increases with the increasing content of CB filler, from 71.0°C for the CB0 sample to 72.7°C for the CB10 sample and 73.8°C for the CB25 sample. This indicates partial reinforcement of the PLA matrix by the CB filler, however, the reinforcing effect is less visible for samples with high content of CB (25 wt.%), which can be explained by the tendency of carbon black to agglomerate and aggregate. Moreover, the peak values of the loss factor decreased for the CB-PLA compared to the CB0 sample, which is related to matrix-filler interactions and internal friction between phases during heating [2].

**S4. The SEM topography analysis of PLA-CB composites with different CB concentration**


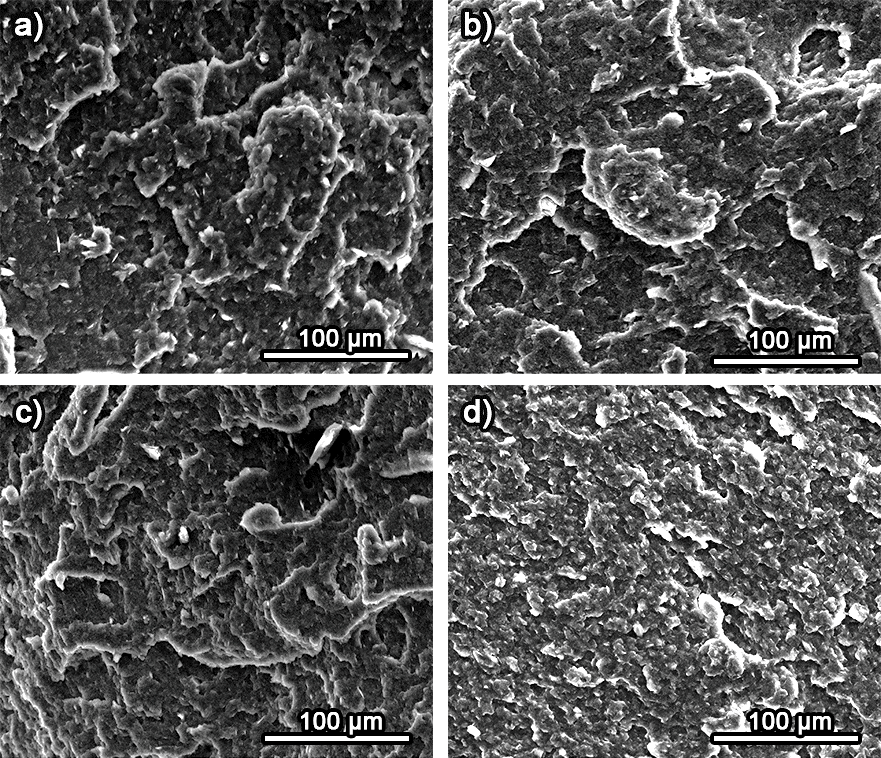


**Figure S4** – SEM micrographs in the cross-section of the filaments formed from CB-PLA composites with different CB content: a) 10%; b) 15%; c) 20%, d) 25%.

**S5. The BET isotherms for different carbon nanofillers used**

**a)**

**b)**

**c)**


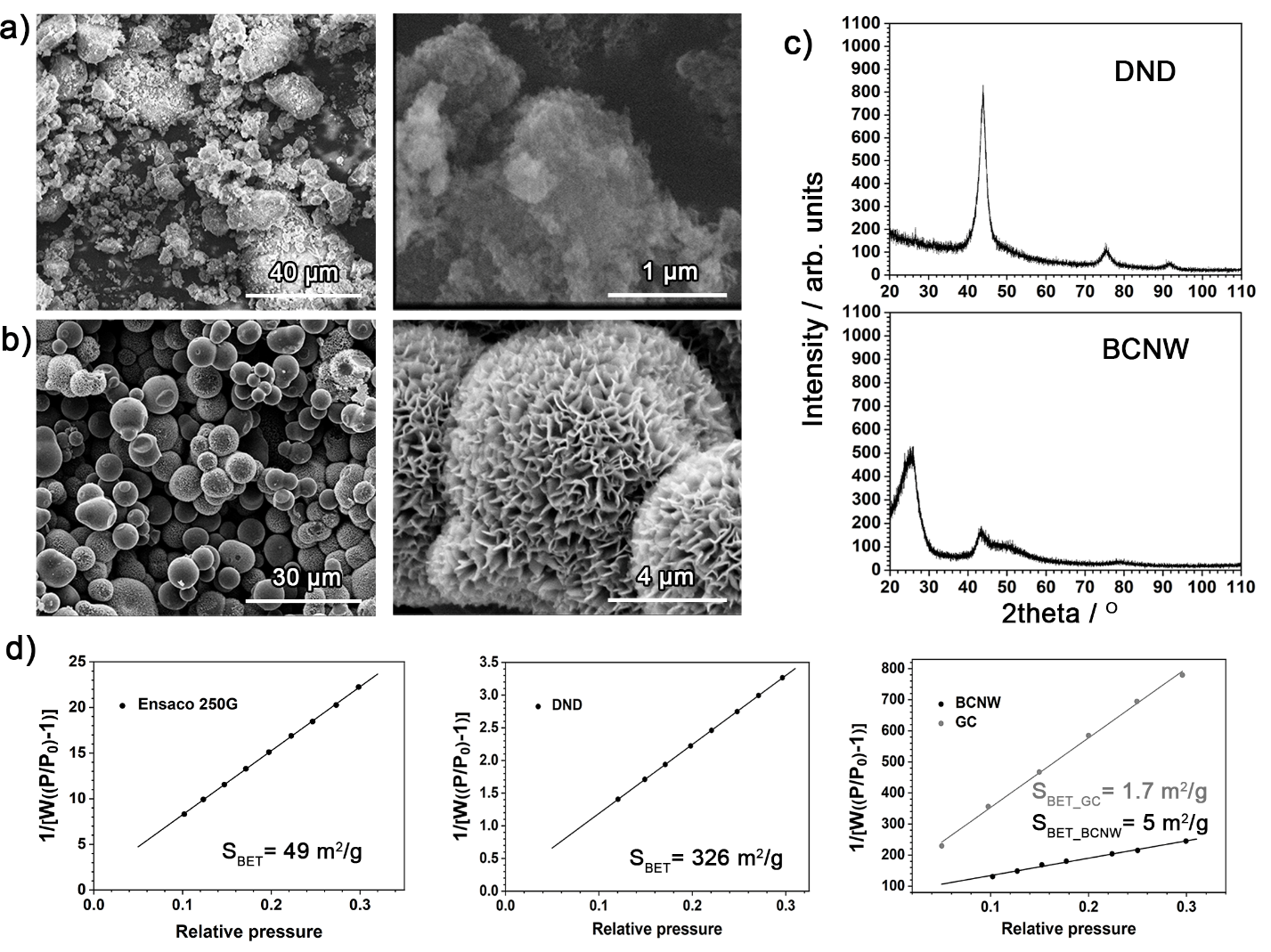


**Figure S5** – The BET isotherms registered for conductive carbon nanopowders: a) CB; b) DND and c) GC and BCNW films grown on GC.

**S6. The topography of GC spheres within the CB20_BCNW5 composite**


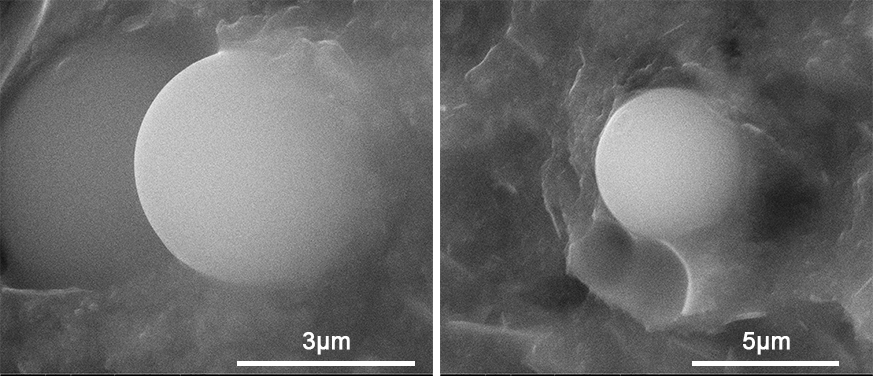


**Figure S6** – SEM micrographs of the GC spheres within CB20_BCNW5 composite.

**S7. Supplementary FTIR and Raman spectroscopy information**


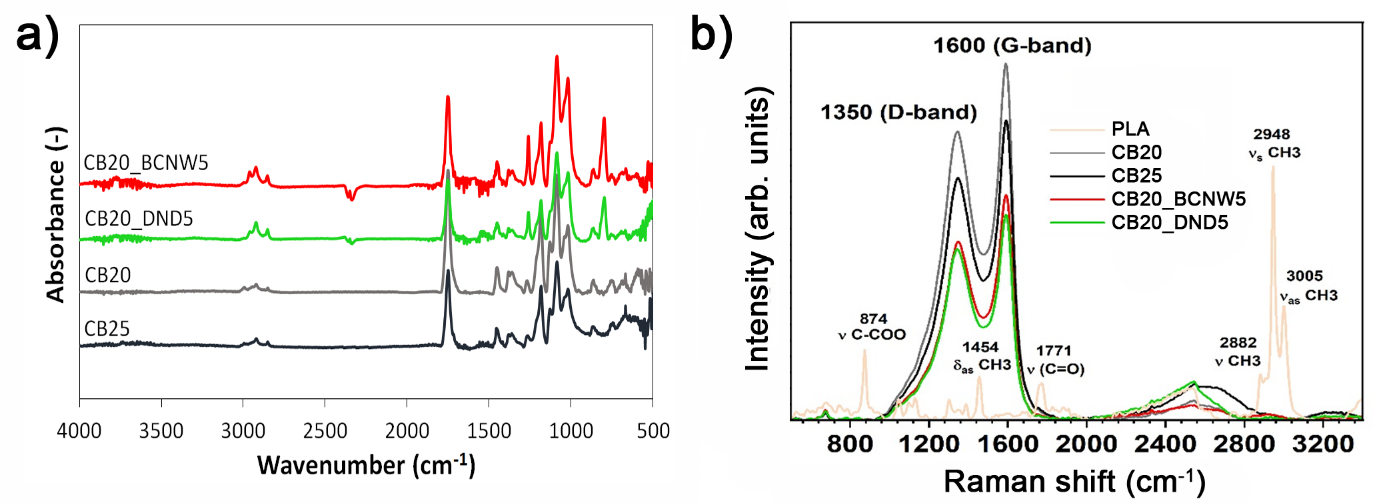


**Figure S7** – Physico-chemical characterisation of CB-DNC-PLA composites with DND and BCNW: a) FTIR and b) Raman spectroscopy studies.

For materials with the addition of DNCs, a new absorption band at about 830 cm^-1^ can be observed, which may correspond to the aromatic =C-H group contained in the structures of the fillers [3]. In these materials, CB20_DND5 in particular, small absorption peaks can also be observed at a wavelength of about 1550 cm^-1^, which further confirms the presence of unsaturated bonds in the DNC structure [4].

The spectra show two main first-order bands located at about 1350 cm^-1^ (D band) and 1600 cm^-1^ (G band) reported for carbon black materials [5] but also being strongly pronounced in both DND [6] and BCNW [7] fillers. It is important to point out that the broad D band can be observed in carbons with high levels of defects, such as CB, and has been assigned to the existence of both non-crystalline carbon and organic molecules containing aliphatic groups attached to the graphitic structure [8]. The broad band observed in the second-order Raman spectra (ranging from 2300 to 2800 cm^-1^) makes it difficult to clearly identify any individual components, such as 2D or D+G. The composite loading of the diamondised nanocarbons was measured qualitatively by estimating the D/G and G/D ratios using spectra deconvolution and area integration. Refer to the **SI file, Table S3** for details.

**Table S3** – D/G peak area ratio from Raman spectroscopy

| **Sample code** | **D/G peak area ratio** | **G/D peak area ratio** |
| --- | --- | --- |
| CB20 | 1.82 | 0.55 |
| CB25 | 1.94 | 0.51 |
| BCNW5 | 1.64 | 0.61 |
| DND5 | 1.72 | 0.58 |

**S8. The summary of the obtained CV results**

**Table S4** – The anodic and cathodic peak currents and peak-to-peak separation based on cyclic voltammetry analysis at 100 mV/s scan rate.

|  | ΔE (mV) | i_p,A_ (µA) | i_p,A_ / \|i_p,C_\| |
| --- | --- | --- | --- |
| CB10 | - | - | - |
| CB15 | - | - | - |
| CB20 | 250 | 79 | 1.3 |
| CB25 | 523 | 131 | 1.45 |
| CB20_BCNW0.5 | 312 | 190 | 1.15 |
| CB20_BCNW5 | 191 | 259 | 1.07 |
| CB20_DND0.5 | 181 | 280 | 1.1 |
| CB20_DND5 | 172 | 312 | 1.27 |

**S9. Electric equivalent circuit deconvolution of the EIS results for various composites**

Electric equivalent circuits were used to estimate the electric parameters characteristic of studied interphases. There is no universal EEC that could be used in this process, the EECs built of one or two time constants (RQ) were used, with constant phase element (CPE) applied instead of capacitance to represent the spatial heterogeneity and the appearance of frequency dispersion of capacitance. Moreover, the Warburg element was used to estimate the diffusion impedance.

It is assumed that the presence of multiple time constants in the case of CB10 and CB15 may originate from a lack of homogeneous distribution of diffusion field due to the absence of a sufficient amount of conductive percolation paths.

For CB-ND-PLA composites a secondary time constant reappears, despite its absence for the CB20 sample, which is presumably due to different rate constants of [Fe(CN)_6_]^3−/4−^ redox process at ND surface (DND or BCNW) and CB surfaces.

**Table S5** – The results of EIS data fitting with an EEC: R(Q(RW)) for samples CB20 and CB25 and R(Q(R(Q(RW)))) for all remaining samples.

|  | **CPE (µFs^α-1^)** | **α (-)** | **R_CT_ (Ω)** | **CPE_2_ (µFs^α-1^)** | **α_2_ (-)** | **R_2_ (Ω)** | **W (µSs^1/2^)** |
| --- | --- | --- | --- | --- | --- | --- | --- |
| **CB10** | 4.27۰10^-4^ | 0.85 | 8.38۰10^4^ | 3.17۰10^-7^ | 0.84 | 5.05۰10^16^ | 6.84۰10^13^ |
| **CB15** | 1.80۰10^-6^ | 0.86 | 21.29 | 2.19۰10^-4^ | 0.19 | 1.79۰10^7^ | 3.57۰10^12^ |
| **CB20** | 3.4۰10^-5^ | 0.80 | 1127 | - | - | - | 9.28۰10^-4^ |
| **CB25** | 3.65۰10^-6^ | 0.81 | 432 | - | - | - | 3.9۰10^-4^ |
| **CB20_BCNW0.5** | 1.27۰10^-5^ | 0.77 | 382 | 2.81۰10^-4^ | 0.91 | 2047 | 2.84۰10^-4^ |
| **CB20_BCNW5** | 3.41۰10^-4^ | 0.74 | 31.4 | 1.4۰10^-4^ | 1.00 | 1153 | 3.11۰10^-3^ |
| **CB20_DND0.5** | 2.43۰10^-4^ | 0.81 | 30.65 | 3.49۰10^-3^ | 0.67 | 1.39۰10^4^ | 9.20۰10^-20^ |
| **CB20_DND5** | 9.31۰10^-5^ | 0.73 | 50.08 | 5.05۰10^-4^ | 0.83 | 699.45 | 2.43۰10^-14^ |

**References:**

1. Guo J, Tsou C-H, Yu Y, et al (2021) Conductivity and mechanical properties of carbon black-reinforced poly(lactic acid) (PLA/CB) composites. Iran Polym J 30:1251–1262. https://doi.org/10.1007/s13726-021-00973-2

2. Zou D, Zheng X, Ye Y, et al (2022) Effect of different amounts of bamboo charcoal on properties of biodegradable bamboo charcoal/polylactic acid composites. International Journal of Biological Macromolecules 216:456–464. https://doi.org/10.1016/j.ijbiomac.2022.06.209

3. Volkov DS, Krivoshein PK, Proskurnin MA (2020) Detonation Nanodiamonds: A Comparison Study by Photoacoustic, Diffuse Reflectance, and Attenuated Total Reflection FTIR Spectroscopies. Nanomaterials 10:2501. https://doi.org/10.3390/nano10122501

4. Shenderova O, Panich AM, Moseenkov S, et al (2011) Hydroxylated Detonation Nanodiamond: FTIR, XPS, and NMR Studies. J Phys Chem C 115:19005–19011. https://doi.org/10.1021/jp205389m

5. Cai N, Dai Q, Wang Z, et al (2014) Preparation and properties of nanodiamond/poly(lactic acid) composite nanofiber scaffolds. Fibers Polym 15:2544–2552. https://doi.org/10.1007/s12221-014-2544-2

6. Mochalin VN, Shenderova O, Ho D, Gogotsi Y (2012) The properties and applications of nanodiamonds. Nature Nanotech 7:11–23. https://doi.org/10.1038/nnano.2011.209

7. Pierpaoli M, Ficek M, Jakóbczyk P, et al (2021) Self-assembly of vertically orientated graphene nanostructures: Multivariate characterisation by Minkowski functionals and fractal geometry. Acta Materialia 214:116989. https://doi.org/10.1016/j.actamat.2021.116989

8. Pawlyta M, Rouzaud J-N, Duber S (2015) Raman microspectroscopy characterization of carbon blacks: Spectral analysis and structural information. Carbon 84:479–490. https://doi.org/10.1016/j.carbon.2014.12.030
